# Measurement of Fall Injury With Health Care System Data and Assessment of Inclusiveness and Validity of Measurement Models

**DOI:** 10.1001/jamanetworkopen.2019.9679

**Published:** 2019-08-21

**Authors:** Lillian Min, Mary Tinetti, Kenneth M. Langa, Jinkyung Ha, Neil Alexander, Geoffrey Hoffman

**Affiliations:** 1 Institute for Healthcare Policy and Innovation, Ann Arbor, Michigan; 2 Geriatric Research Education Clinical Center, Virginia Ann Arbor Healthcare System, Ann Arbor, Michigan; 3 Division of Geriatric and Palliative Medicine, Department of Internal Medicine, University of Michigan, Ann Arbor; 4 Section of Internal Medicine, Department of Medicine, Yale School of Medicine, New Haven, Connecticut; 5 Division of General Medicine, Department of Internal Medicine, University of Michigan, Ann Arbor; 6 Institute for Social Research, University of Michigan, Ann Arbor; 7 University of Michigan School of Nursing, Ann Arbor

## Abstract

**Importance:**

National injury surveillance systems use administrative data to collect information about severe fall-related trauma and mortality. Measuring milder injuries in ambulatory clinics would improve comprehensive outcomes measurement across the care spectrum.

**Objectives:**

To assess a flexible set of administrative data–only algorithms for health systems to capture a greater breadth of injuries than traditional fall injury surveillance algorithms and to quantify the algorithm inclusiveness and validity associated with expanding to milder injuries.

**Design, Setting, and Participants:**

In this longitudinal diagnostic study of 13 939 older adults (≥65 years) in the nationally representative Health and Retirement Study, a survey was conducted every 2 years and was linked to hospital, emergency department, postacute skilled nursing home, and outpatient Medicare claims (2000-2012). During each 2-year observation period, participants were considered to have sustained a fall-related injury (FRI) based on a composite reference standard of having either an external cause of injury (E-code) or confirmation by the Health and Retirement Study patient interview. A framework involving 3 algorithms with *International Classification of Diseases, Ninth Revision* codes that extend FRI identification with administrative data beyond the use of fall-related E-codes was developed: an acute care algorithm (head and face or limb, neck, and trunk injury reported at the hospital or emergency department), a balanced algorithm (all acute care algorithm injuries plus severe nonemergency outpatient injuries), and an inclusive algorithm (almost all injuries). Data were collected from January 1, 1998, through December 31, 2012, and statistical analysis was performed from August 1, 2016, to March 1, 2019.

**Main Outcomes and Measures:**

Validity, measured as the proportion of potential FRI diagnoses confirmed by the reference standard, and inclusiveness, measured as the proportion of reference-standard FRIs captured by the potential FRI diagnoses.

**Results:**

Of 13 939 participants, 1672 (42.4%) were male, with a mean (SD) age of 77.56 (7.63) years. Among 50 310 observation periods, 9270 potential FRI diagnoses (18.4%) were identified; these were tested against 8621 reference-standard FRIs (17.1%). Compared with the commonly used method of E-coded–only FRIs (2-year incidence, 8.8% [95% CI, 8.6%-9.1%]; inclusion of 51.5% [95% CI, 50.4%-52.5%] of the reference-standard FRIs), FRI inclusion was increased with use of the study framework of algorithms. With the acute care algorithm (2-year incidence, 12.6% [95% CI, 12.4%-12.9%]), validity was prioritized (88.6% [95% CI, 87.4%-89.8%]) over inclusiveness (62.1% [95% CI, 61.1%-63.1%]). The balanced algorithm showed a 2-year incidence of 14.6% (95% CI, 14.3%-14.9%), inclusion of 65.3% (95% CI, 64.3%-66.3%), and validity of 83.2% (95% CI, 81.9%-84.6%). With the inclusive algorithm, the number of potential FRIs increased compared with the E-code–only method (2-year incidence, 17.4% [95% CI, 17.1%-17.8%]; inclusion, 68.4% [95% CI, 67.4%-69.3%]; validity, 75.2% [95% CI, 73.7%-76.6%]).

**Conclusions and Relevance:**

The findings suggest that use of algorithms with *International Classification of Diseases, Ninth Revision* codes may increase inclusion of FRIs by health care systems compared with E-codes and that these algorithms may be used by health systems to evaluate interventions and quality improvement efforts.

## Key Points

**Question:**

What are the tradeoffs between inclusiveness and validity when expanding the external mechanism-of-injury codes (E-codes) for fall-related injury to include *International Classification of Diseases, Ninth Revision* (*ICD-9*)–coded injuries in older adults?

**Findings:**

In this diagnostic study of 13 939 older adults (≥65 years) in the nationally representative Health and Retirement Study, an external reference standard of patient interview (E-codes) was used as evidence that a fall caused the injury. With use of hospital and emergency department *ICD-9* codes, the acute care algorithm maximized validity and the inclusive algorithm prioritized milder injuries seen in outpatient clinic care, doubling the number of fall-related injuries captured compared with E-codes alone.

**Meaning:**

The findings suggest that health care systems can increase inclusion of fall-related injury by adding *ICD-9–*coded injuries to the existing method of using only fall-related E-codes.

## Teaser

This diagnostic study uses Health and Retirement Survey and linked Medicare data to develop and validate an administrative approach to identifying older adults with fall-related injuries and to quantify the inclusiveness and validity across a spectrum of potential diagnoses of fall-related injuries.

## Introduction

Falls among older adults are associated with serious morbidity, mortality, and cost^1,2,3,4^ but are preventable.^5^ From a population health perspective, fall surveillance can bolster prevention efforts by targeting high-risk individuals.^6^ However, existing surveillance systems rely heavily on external cause of injury codes (E-codes) which are used by the US trauma health care system to determine that an injury was caused by a fall (as opposed to another traumatic cause).^7,8,9,10^ While accurate, this injury identification approach is limited in nonemergency outpatient clinic settings where E-codes are not required and hampers efforts to gain a complete picture of fall-related injury (FRI).

As health care systems develop fall injury reduction programs, monitoring of programs' effectiveness requires surveillance of both severe and moderate injuries (ie, including those in which a fall E-code was not used but instead involving diagnostic codes in claims data only). There are important tradeoffs involved in broadening surveillance systems beyond the use of only E-codes. On the one hand, such injuries as hip fracture and head trauma are likely to be FRIs because falls are the most common cause of severe, traumatic injury among older adults.^7,8,9,10^ The inclusion of mild injuries in surveillance systems may reduce the validity of those FRI diagnoses that are identified because mild injuries may be due to nonfall causes. On the other hand, including mild injury (eg, sprains and contusions) would favorably increase the power to detect intervention associations, which depended heavily on the rate of outcomes. In addition, mild injuries are important causes of pain and accelerated disability in older people.^11,12^ The inclusion of mild injuries in surveillance systems may reduce the validity of the FRI diagnoses while increasing the total number of FRIs identified because not all mild injuries are fall related.

Earlier research involving fall surveillance has extended claims-based diagnoses beyond E-codes without explicitly considering tradeoffs of inclusiveness and validity.^12,13,14,15,16^ We enhanced this work by examining inclusiveness and validity across a spectrum of potential inpatient and outpatient FRI diagnoses.

## Methods

### Data

This longitudinal diagnostic study used Health and Retirement Study (HRS) Core and Exit (postmortem) surveys (biennially from January 1, 2000, through December 31, 2012) and linked Medicare claims from inpatient hospital, ambulatory (emergency department and outpatient clinics), and nursing home claims files from January 1, 1998, through December 31, 2012. We included older (age ≥65 years) HRS respondents who consented to providing their Medicare beneficiary numbers for linking their interviews to fee-for-service Medicare (Parts A and B) claims data. The study was approved by the University of Michigan institutional review board; informed consent was waived because this study was a secondary analysis of deidentified data. Data were collected from January 1, 1998, through December 31, 2012, and statistical analysis was performed from August 1, 2016, to March 1, 2019. The study followed the Standards for Reporting of Diagnostic Accuracy (STARD) reporting guideline.

### Reference Standard Used for Validating FRIs

To develop a set of *International Classification of Diseases, Ninth Revision* (*ICD-9*) FRI diagnoses beyond fall-related E-codes, patient-reported FRIs (indicating an injurious fall in the previous 2 years) were added to E-coded FRIs (eTable 1 in the Supplement) to create a composite of the 2 FRI identifiers. A composite reference standard is 1 approach to evaluating a new diagnostic test in the absence of an external criterion standard.^17^ In a data set of this size and scope, a large sample of *ICD-9* codes were tested against both internal (E-codes) and external (interview) data validation sources. By adopting this next best reference standard, test properties were calculated and they prioritized the injury codes by validity. Because this *ICD-9–*based algorithm was intended to measure FRIs in future practice, the primary criterion for prioritizing *ICD-9* codes as more valid was higher positive predictive value (proportion of tests events confirmed by the reference standard). Thus, further references to validity in this report were synonymous to positive predictive value. Validity was operationalized as a tradeoff with inclusiveness (proportion of the reference standard captured by the test, synonymous to sensitivity) as the scope of injuries considered to include milder injuries was broadened. This approach was only possible by considering the reference standard, even if imperfect, as a true measure of FRI.

### Injury Diagnoses to Be Tested

Using all claims from Medicare hospital, nursing home, and outpatient (emergency department and clinic) encounters, we identified all injuries (*ICD-9* diagnosis codes 800-999) excluding burns, splinters, bites, and fractures associated with metastatic bone disease. The remaining diagnoses were considered as potential FRIs to be tested against the reference standard. Next, we created a matrix of potential FRI categories consisting of 3 injury types by 4 anatomic regions. Injury types were bone fractures, sprains, strains, dislocations, and superficial skin injuries. Regions were the head, neck or trunk, upper limb, and lower limb. Finally, a nonspecific and internal injury category was created (eTable 2 and eTable 3 in the Supplement).

To identify potential FRIs treated exclusively in emergency department and outpatient clinics, a UCLA/RAND algorithm^16^ was extended that requires a casting or splinting procedure on the same day or an imaging study in less than 10 days. We further included superficial injuries such as bruises and abrasions as potential FRIs (eTables 4-8 in the Supplement) and added suturing to these inclusion criteria.

**Figure 1.**
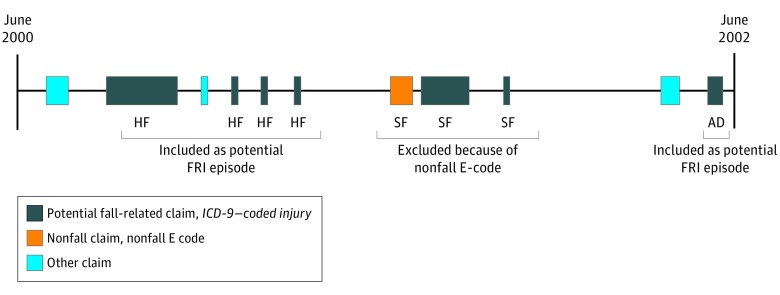
Construction of Fall-Related Injury (FRI) Episodes of Care From Claims Claims for external cause of injury–coded (E-coded) reference standard or *International Classification of Diseases, Ninth Revision (ICD-9)–*coded potential FRIs were grouped into episodes of care during 2-year observation windows (the unit of analysis) before the interviews. Left, a potential FRI episode, an initial hip fracture (HF) *ICD-9* code, and multiple subsequent same-injury diagnoses for follow-up care within 180 days. Middle, a skull fracture (SF) *ICD-9* was excluded because of an E-code for nonfall (motor vehicle crash). Right, a single diagnosis code indicates a potential FRI involving an ankle dislocation (AD).

Potential FRIs were grouped into episodes, with claims across health care settings that occurred within fewer than 180 days in the same anatomic region and injury type combined into single episodes of care (Figure 1); this served as a look-back period to prevent preexisting injury from counting as a new event. Potential FRI episodes beginning with a non-FRI E-code (eg, motor vehicle crash) were excluded.

### Statistical Analysis

Potential FRI episodes occurring during the 2 years preceding each HRS survey interview were identified (ie, corresponding to the period covered by the interview question). These 2-year observation windows were the unit of analysis for testing whether potential FRI episodes were validated by the reference standard, (ie, if a reference standard FRI was present during the same period). The earliest date within the FRI episode was used to determine which observation window to use (Figure 1). The use of these episodes ensured that codes appearing multiple times in claims over time (ie, for follow-up care of the same injury) would not count as a new injury in the next observation period. Further details (along with annotated SAS code [SAS, version 9.4; SAS Institute]) are provided in eTables 4-8 in the Supplement.

Validity of each category of *ICD*-coded injury diagnoses was calculated by ascertaining the copresence of a reference-standard FRI with the test FRI episode within the same observation window (Figure 1) (Table 1). Then, using these true-positives (number of test FRI episodes confirmed by the reference standard) as the numerator, we divided by all the potential FRI episodes (the denominator; ie, all the test-positives). In this initial descriptive analysis, validity was calculated within each of the consolidated categories of potential FRIs.

**Table 1.** Composite Reference Standard for FRIs vs Potential FRIs in 2-Year Observation Windows

Level of Health Care for All Potential *ICD-9–*Coded FRIs	Reference Standard, Cases, No. (%) (N = 50 310)			
	Only E-coded FRI	Only Self-reported FRI	Both E-coded and Self-reported FRI	Neither E-coded nor Self-reported FRI
Acute hospital or postacute nursing home care for >3-d stay (n = 2264)^a^	295^b^ (13.0)	432^b^ (19.1)	1013^b^ (44.7)	524^c^ (23.1)
Any emergency department visit, hospital, or postacute nursing home care, any length of stay (n = 5836)^a^	1141^b^ (19.6)	1023^b^ (17.5)	2155 ^b^ (36.9)	1517^c^ (26.0)
All levels of inpatient or outpatient care (n = 9270)^a^	1425^b^ (15.4)	1617 ^b^ (17.4)	2379^b^ (25.7)	3849^c^ (41.5)
No potential *ICD-9* test FRIs (n = 41 040)	400^d^ (1.0)	2566^d^ (6.3)	234^d^ (0.6)	37 840^e^ (92.2)

Abbreviations: E-code, external cause of injury code; FRIs, fall-related injuries; *ICD-9*, *International Classification of Diseases, Ninth Revision*.

^a^ Row 1 is nested in row 2, which is nested in row 3.

^b^ True-positives.

^c^ False-positives.

^d^ False-negatives.

^e^ True-negatives.

To explore differences in validity by level of health care acuity, potential FRI episodes were stratified by presence of any acute hospital data (total hospital plus any postacute nursing home care ≥3 days), any emergency department or short hospital stay (<3 days), and outpatient clinic only data.

Within each level of health care acuity, injury categories were consolidated to simplify the matrix of injuries and reduce categories with low sample sizes. We screened the least-valid categories (in which <60% of potential FRIs were validated) for outlier diagnoses with poor validity (in which <50% were validated). These diagnoses were removed from their original categories and placed into a distinct, generalized category of nonvalidated injuries.

The results were used to develop a framework involving 3 algorithms that extend FRI identification with administrative data beyond the use of FRI E-codes: (1) an acute care algorithm identifying the most valid and severe injuries, (2) a balanced algorithm occupying a middle ground in validity and inclusion, and (3) an inclusive algorithm, which maximizes inclusion of FRIs found across the health care system. To develop the framework, we sorted the consolidated injury categories from the most to the least valid within each health care acuity level and sequentially included the categories according to their validity. In this way, we quantified the loss in validity (and gain in inclusiveness) as additional, less-valid FRI categories were added. Specifically, E-coded FRIs were the first category to be included in each algorithm because they were, by definition, the most valid administrative data (100% valid because they were part of the reference standard). Then, beginning with acute care settings, we sequentially added the next most valid of the potential FRI categories to E-coded FRIs, reporting the loss in validity and increase in inclusiveness of the resulting cumulative FRI algorithm at each step.

These steps were then sequentially repeated for consolidated FRI categories across less acute health care settings until all data were considered in the algorithm framework. To address known recall bias resulting from interviewing patients about FRIs,^18,19,20,21,22^ validity of the final algorithms was presented among the FRIs less than 6 months before the interviews.

## Results

### Descriptive Results

There were 20 334 participants (aged ≥65 years) in the HRS who participated in 83 769 biennial interviews from 2000 through 2012. Of these, 13 939 individuals allowed HRS to link their survey data with fee-for-service Medicare data, resulting in a final analytic sample of 50 310 two-year observation windows. The sample of 13 939 participants was 42.4% male (1672 participants), with a mean (SD) age of 77.56 (7.63) years.

In the Medicare data, more than 2.8 million claims across all levels of acuity were identified, of which 0.68% included E-coded FRIs and 1.85% included *ICD-9*–coded potential FRIs (Table 2). After we linked E-coded FRI encounters into episodes, 4438 (8.8%) were found in 50 310 unique 2-year observation windows. After including patient-reported interviews, the number of observation windows containing a reference standard FRI increased to 8621 (17.1% of observations). Using 52 060 eligible *ICD-9* diagnoses (Table 2), we constructed 9270 potential FRI episodes (18.4% of 50 310 observation windows) to test against the reference standard FRIs (Table 1).

**Table 2.** Administrative Data Sources of Injuries Among Older US Adults, 1998-2012^a^

Description of Data	Claims, No. (%) (N = 2.8 Million)
Data source for all health care encounter claims	
Hospital	40 120 (1.4)
Nursing home	15 906 (0.6)
Emergency department	33 818 (1.2)
Outpatient clinic	2 731 571 (96.8)
E-coded fall-related injuries	
Motor vehicle crash	803 (0)
Accidental falls	9685 (0.3)
Other	8642 (0.3)
Potential FRIs tested	
Head and face	
Fracture of skull and head trauma	2391 (0.1)
Open wound, superficial injury, or contusion	4618 (0.2)
Dislocation of jaw	6 (0)
Sprains and strains	61 (0)
Injury to blood vessels or nerve, crushing injury	29 (0)
Neck and trunk	
Fracture	5358 (0.2)
Open wound, superficial injury, or contusion	2173 (0.1)
Dislocation	424 (0)
Sprains and strains	2084 (0.1)
Internal injury	378 (0)
Injury to blood vessels or nerve, crushing injury	158 (0)
Upper extremity	
Fracture	10 135 (0.4)
Open wound, superficial injury, or contusion	3557 (0.1)
Dislocation	630 (0)
Sprains and strains	1638 (0.1)
Injury to blood vessels or nerve, crushing injury	54 (0)
Lower extremity	
Fracture	17 324 (0.6)
Open wound, superficial injury, or contusion	4054 (0.1)
Dislocation	1316 (0.1)
Sprains	2570 (0.1)
Injury to blood vessels or nerve, crushing injury	58 (0)

Abbreviations: E-code, external cause of injury code; FRIs, fall-related injuries.

^a^ Medicare injury encounter data for participants in the 1998-2012 Health and Retirement Study before consolidating E-codes and potential FRIs claims into episodes of care. There were 19 130 E-coded FRIs (0.68% of all claims) and 52 060 potential FRIs tested (1.85% of all claims).

### Validity in High-Acuity Care Settings

**Figure 2.**
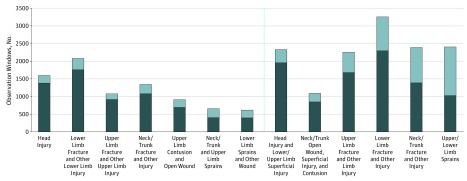
Consolidated Categories of Potential Fall-Related Injury (FRI) Episodes by Sample Size and Validity Prevalence (entire bar) and validity (dark gray) of consolidated groups of potential FRI *International Classification of Diseases, Ninth Revision (ICD-9)*–coded test episodes before testing in the algorithms. Cases are number of 2-year observation periods containing episodes. Left, FRI episodes involving care in acute settings (hospital, emergency department, and postacute nursing facilities). Right, all inpatient and outpatient settings. Each category is considered independently from the others; the same observation window can be included more than once.

The overall validity of *ICD-9* injuries was similar in the 2 high-acuity care categories, and therefore the categories were combined (any hospital, nursing home, or emergency department care). After consolidating injury types, the most valid injury category from higher-acuity care settings (Figure 2) was head and face injuries (1602 FRI episodes; 86.0% validity).

### Validation Across All Inpatient and Outpatient Health Care Settings

Among all potential FRIs (Figure 2), the most valid category remained head and face injury (2332 episodes; 83.7% validity), whereas the invalid injury category (injuries with <50% validity) consisted of upper and lower extremity sprains (2405 episodes; 42.7% validity).

Most superficial injuries (contusions, lacerations, and abrasions) were validated at more than 70%. A post hoc sensitivity analysis (eTable 9 in the Supplement) showed that this high validity was not a function of superficial injuries occurring during the same observation window as more severe injuries.

### Validity and Inclusiveness of Cumulative Diagnosis-Based Algorithms

**Figure 3.**
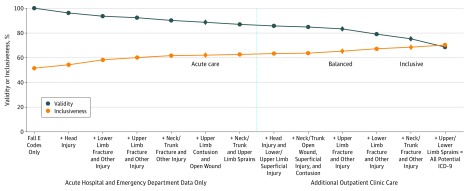
Inclusiveness vs Validity in 3 Fall-Related Injury (FRI) Administrative Data Algorithms Cumulative result of adding test *International Classification of Diseases, Ninth Revision (ICD-9)*–coded injuries to algorithm for inclusiveness and validity. Left of dotted line, E-coded FRIs with additions until all potential *ICD-9*–coded injuries are included into the test algorithm. Inclusion indicates percentage of reference-standard observation windows (9904 periods) captured by the algorithm up to that point. Validity indicates proportion of test FRIs within 6 months of an interview up to that point (an increasing denominator, from 0 test FRIs until the maximum of 3936 observation periods) validated by the reference standard.

Our final framework of 3 nested FRI algorithms revealed trade-offs between validity and inclusiveness (Figure 3). For comparison, as a baseline reference, each algorithm was compared with one only including E-coded FRIs (2-year incidence, 8.8% [95% CI, 8.6%-9.1%]; validity, 100%; inclusiveness, 51.5% [95% CI, 50.4%-52.5%]). The acute care algorithm (Figure 3) revealed an incidence of FRIs of 12.6% (95% CI, 12.4%-12.9%) and a 43% increase in test-positive cases compared with E-coded FRIs only, the baseline reference. The acute care algorithm was 88.6% (95% CI, 87.4%-89.8%) valid and 62.1% (95% CI, 61.1%-63.1%) inclusive (a 21% increase).

The balanced algorithm revealed an incidence of FRIs of 14.6% (95% CI, 14.3%-14.9%), which was a 65.7% increase compared with E-codes only, and was 83.2% (95% CI, 81.9%-84.6%) valid and 65.3% (95% CI, 64.3%-66.3%) inclusive (a 27% increase compared with E-codes only).

The inclusive algorithm, which extends beyond hospitals and emergency department care into outpatient clinic care, revealed an incidence of FRIs of 17.4% (95% CI, 17.1%-17.8%), which was a 98.2% increase compared with E-codes only, and was 75.2% (95% CI, 73.7%-76.6%) valid and 68.4% (95% CI, 67.4%-69.3%) inclusive (a 32.8% increase compared with E-codes only).

Validity of all 3 algorithms was improved with use of the UCLA/RAND method (requiring procedure codes as inclusion criteria to identify FRIs exclusively diagnosed in outpatient emergency departments and clinics), with the greatest improvement observed for the inclusive algorithm (from 53.2% to 75.2%) (eTable 10 in the Supplement).

The category of low-validity injuries (upper and lower limb sprains from both inpatient and outpatient care settings) was not included in any of the 3 final algorithms. Adding these remaining injuries would have identified more cases (19.7% incidence and 70.2% inclusiveness) but would have reduced validity (68.6%) (Figure 3).

## Discussion

In this study, we developed and validated an administrative approach using injury diagnostic data sets from across health settings to identify older adults with FRIs ranging from high to low acuity, improving on existing methods that rely on E-codes for detection of fall-related health care. The result was a flexible framework of algorithms that traded off in validity and inclusiveness, with implications for surveillance and quality improvement measurement. Using only high-acuity FRIs, the acute care algorithm was the most valid but least inclusive, with 88.6% of its test FRIs confirmed by the reference standard and approximately 62.1% of all reference-standard FRIs captured by test FRIs. By examining outpatient clinical care diagnoses, the inclusive algorithm had a 32.8% increase in reference-standard FRIs captured by test FRIs with a decrease in validity (from 90% to 75%). Together, these results suggest that by balancing validity with inclusiveness, FRIs of varying severity can be broadly identified using commonly used administrative databases that go beyond the use of E-codes.^23^

Previous work^7,11,24,25,26,27^ used FRI algorithms that were limited to only E-coded injuries, patient interview, or medical record review (limited by cost). In comparison with previous algorithms,^13,14,15,16^ we developed more comprehensive algorithms, including less severe injuries (abrasions, contusions, and lacerations) while removing less valid injury diagnoses. This was achieved by leveraging the thousands of injuries in Medicare data that were validated against E-codes and interviews from the nationally representative HRS—a process not possible in other data sets. The large sample of injuries also allowed us to identify injury categories within health care acuity levels to refine the algorithms. This study was also the first, to our knowledge, to offer its algorithm codes for public use.

We propose that this algorithm framework can facilitate assessment of interventions^28,29,30^ to reduce fall risk among individuals^31,32,33^and within communities.^34,35,36,37,38^ The acute care algorithm identified 43% more FRIs compared with the E-code only approach while maintaining strong validity; thus, it may be a valid tool for increasing statistical power to detect significant changes resulting from an intervention (eg, a resource-intensive polypharmacy review to reduce fall risk). Because of its broader scope but reduced validity, the balanced algorithm can serve to identify fall outcomes for lower-risk quality improvement initiatives in primary care, such as promoting use of the Medicare annual medical wellness visit or for improving care of older persons who fall.^39,40^ The inclusive algorithm was the most comprehensive approach (doubling the number of FRIs compared with E-codes only) and included more superficial injuries. By excluding the least-valid injuries while maintaining most other injuries, it captured the most FRIs of the 3 algorithms and therefore may be the most useful for public health monitoring. For individuals who are more frail, this measure captures even the minor injuries that lead to fear of falling and loss of independence.

Each of these surveillance tools can contribute to ongoing efforts in fall prevention. For population-based efforts^41,42,43,44^ and clinical practice improvement efforts to reduce falls^45,46,47,48,49^ to work, valid tools to measure their effectiveness will be required. However, although large health care systems are increasingly responsible for monitoring quality and outcomes, they often lack the tools to do so. Medicare also does not have critical population-wide fall surveillance for older adults, although falls are one of the leading causes of death among older adults.^3,7,50^ Instead, present approaches to monitoring fall injuries and their costs,^1,12,14,15,24,51^ including data from the US trauma system,^52^ focus on fall-related death and more severe injury and ignore falls treated in outpatient clinics, which represent a large proportion of older adult falls. Our algorithms can be considered a step toward addressing these limitations and improve monitoring.

As increasing attention is paid to falls in an aging US population, one direction is to capture clinical events in electronic health records. Next steps include developing methods to capture falls reported by patients by telephone, email, or electronic visits either by requiring diagnoses for these alternative means of accessing health care or by using natural language processing to read clinical notes.^53^ In addition, triangulating diagnostic codes with both patient report and clinician notes would be a resource-intensive but rigorous way to further develop an administrative algorithm for FRI identification.^49^ These approaches may not be as broadly generalizable as our present claims-based approach given the intersystem differences in electronic health record use, type, and data availability but may be a step toward more accurate monitoring of falls.

### Limitations

We noted 3 limitations. First, because of the HRS design, we could only examine patient reports of FRIs every 2 years. Instead of being contemporaneous, patient report of fall injuries involved a 2-year recall period. Previous research suggested that accuracy of recall using falls calendars declined after 6 months^18,19,21,22^ and a modest decline in reporting accuracy 1 year after an FRI. In the present analysis, we used relative ranking of injury types to develop the algorithms. Given that the injury types were randomly distributed in time, the 2-year time frame should not bias our results. Second, use of *International Statistical Classification of Diseases and Related Health Problems, Tenth Revision (ICD-10)* diagnosis codes became mandatory as of 2015, with expansion of both cause of injury and diagnostic injury codes. We believe that these changes will not increase the validity of reported FRIs because there have been no concomitant changes in the mandates for reporting of falls by clinicians. To facilitate future use of the algorithms, we provided proposed *ICD-10* codes corresponding to our *ICD-9*–based algorithms (eTable 11 in the Supplement). Further research is needed to revalidate the *ICD-10* algorithms. Third, the validity of our algorithms may be biased downward because of unreported FRIs occurring within nursing homes and hospitals. One future improvement would be to identify additional reference-standard FRIs in the minimum data set given standard reporting requirements for falls that are mandatory in nursing facilities.

## Conclusions

The findings suggest that fall injury data should be collected broadly in health systems and for use in public health monitoring by leveraging both the validity of E-codes and *ICD* codes for injuries. By accounting for variability in the validity but also the contribution to overall inclusiveness when adding *ICD* codes, this research organized the codes into algorithms that can be tailored to the measurement needs of health systems.

## Notes

Supplement. **eTable 1.** E Codes Indicating Fall as Cause of Injury **eTable 2.** Matrix of Potential Fall Injuries: *ICD-9* Codes by Anatomical Parts and Potential Type of Fall Injury **eTable 3.** Final Consolidated Injury Groups **eTable 4.** Coding Appendices Overview **eTable 5.** SAS Code to Analyze Medicare Claims Eligible for Potential Fall Injury **eTable 6.** SAS Code for the Fall Algorithm 1 (Acute Care) **eTable 7.** SAS Code for the Fall Algorithm 2 (Balanced) **eTable 8.** SAS Code for Fall Algorithm 3 (Inclusive Algorithm) **eTable 9.** Sensitivity Analysis for Superficial Injuries **eTable 10.** Three Algorithm Validity With and Without Using *CPT* Code Criteria **eTable 11.** Link to Replace *ICD-9* Codes in All 3 Algorithms to *ICD-10*
