# Error in Byline

**DOI:** 10.1001/jamanetworkopen.2019.13086

**Published:** 2019-09-20

In the Original Investigation titled “Measurement of Fall Injury With Health Care System Data and Assessment of Inclusiveness and Validity of Measurement Models,”^1^ published August 21, 2019, in the byline, the middle initial was missing for Geoffrey J. Hoffman, PhD. This article has been corrected.^1^
