# Supplementary material for: Measurement of Fall Injury With Health Care System Data and Assessment of Inclusiveness and Validity of Measurement Models
Source: JAMA Netw Open. 2019 Aug 21;2(8):e199679. doi: 10.1001/jamanetworkopen.2019.9679 (PMC6707014; doi:10.1001/jamanetworkopen.2019.9679)
Supplement: Supplement. — eTable 1. E Codes Indicating Fall as Cause of Injury eTable 2. Matrix of Potential Fall Injuries: ICD-9 Codes by Anatomical Parts and Potential Type of Fall Injury eTable 3. Final Consolidated Injury Groups eTable 4. Coding Appendices Overview eTable 5. SAS Code to Analyze Medicare Claims Eligible for Potential Fall Injury eTable 6. SAS Code for the Fall Algorithm 1 (Acute Care) eTable 7. SAS Code for the Fall Algorithm 2 (Balanced) eTable 8. SAS Code for Fall Algorithm 3 (Inclusive Algorithm) eTable 9. Sensitivity Analysis for Superficial Injuries eTable 10. Three Algorithm Validity With and Without Using CPT Code Criteria eTable 11. Link to Replace ICD-9 Codes in All 3 Algorithms to ICD-10 [file jamanetwopen-2-e199679-s001.pdf]

Supplementary Online Content

Min L, Tinetti M, Langa KM, Ha J, Alexander N, Hoffman G. Measurement of fall injury with health care system data and assessment of inclusiveness and validity of measurement models. *JAMA Netw Open*. 2019;2(8):e199679. doi:10.1001/jamanetworkopen.2019.9679

eTable 1. E Codes Indicating Fall as Cause of Injury
eTable 2. Matrix of Potential Fall Injuries: *ICD-9* Codes by Anatomical Parts and Potential Type of Fall Injury
eTable 3. Final Consolidated Injury Groups
eTable 4. Coding Appendices Overview
eTable 5. SAS Code to Analyze Medicare Claims Eligible for Potential Fall Injury
eTable 6. SAS Code for the Fall Algorithm 1 (Acute Care)
eTable 7. SAS Code for the Fall Algorithm 2 (Balanced)
eTable 8. SAS Code for Fall Algorithm 3 (Inclusive Algorithm)
eTable 9. Sensitivity Analysis for Superficial Injuries
eTable 10. 3 Algorithm Validity With and Without Using CPT Code Criteria
eTable 11. Link to Replace *ICD-9* Codes in All 3 Algorithms to *ICD-10*

This supplementary material has been provided by the authors to give readers additional information about their work.

© 2019 Min L et al. *JAMA Network Open.*

eTable 1. E Codes Indicating Fall as Cause of Injury

E code	Inclusion
E880 Accidental fall on or from stairs or steps	X
E881 Accidental fall on or from ladders or scaffolding	
E882 Accidental fall from or out of building or other structure	
E883 Accidental fall into hole or other opening in surface	
E884 Other accidental falls from one level to another	
E884.0 Accidental fall from playground equipment	X
E884.1 Accidental fall from cliff	
E884.2 Accidental fall from chair	X
E884.3 Accidental fall from wheelchair	X
E884.4 Accidental fall from bed	X
E884.5 Accidental fall from other furniture	X
E884.6 Accidental fall from commode	X
E884.9 Other accidental fall from one level to another	X
E885 Accidental fall on same level from slipping tripping or stumbling	
E885.0 Fall from (nonmotorized) scooter	
E885.1 Fall from roller skates	
E885.2 Fall from skateboard	
E885.3 Fall from skis	
E885.4 Fall from snowboard	
E885.9 Fall from other slipping, tripping, or stumbling	X
E886 Fall on same level from collision, pushing, or shoving, by or with other person	
E887 Fracture, cause unspecified	X
E888 Other and unspecified fall	X

© 2019 Min L et al. *JAMA Network Open.*

eTable 2. Matrix of Potential Fall Injuries: *ICD-9* Codes by Anatomical Parts and Potential Type of Fall Injury

By anatomical parts & injury types	ICD 9 codes	Acute care (hospital, emergency room, and post-hospital nursing home) only					All injuries (inpatient and outpatient care)				
		Group ID	Number of observational periods		SEN	PPV	Group ID	Number of observational periods		SEN	PPV
			Any code	Any code validated for FRI				Any code	Any code validated for FRI		
			5836	4315	50.08	73.94		9270	5416	62.86	58.42
**Head and face**											
Fracture of skull and head trauma	800.xx-804.xx, 829.xx, 850.xx-854.xx	1	393	329	3.82	83.72	7	776	608	7.06	78.35
Open wound, Superficial injury, Contusion	870.xx-872.6x (excluding xxx.1, 871.5, 871.6), 872.8, 873.0, 873.2x, 873.4x, 873.6x, 873.73, 873.8, 910.0, 910.8, 918.0, 918.9, 920.xx-921.xx, 924.8, 924.9		1383	1202	13.94	86.91		1755	1517	17.60	86.44
Dislocation of jaw	830.xx, 839.8, 839.9		1	1	0.01	100.00		3	1	0.01	33.33
Sprains and strains	848.0, 848.1, 848.2, 848.8, 848.9		9	7	0.08	77.78		21	20	0.23	95.24
Injury to blood vessels/nerve, Crushing injury	900.xx, 925.xx, 950.xx, 951.xx		2	0	0.00	0.00		22	18	0.21	81.82
**Neck and trunk**											

© 2019 Min L et al. *JAMA Network Open.*

Fracture	805.xx-806.xx, 807.0x-807.1x, 807.2-807.6, 808.xx, 809.xx, 829.xx		798	637	7.39	79.83	11	1454	1013	11.75	69.67
Open wound, Superficial injury, Contusion	874.0x, 874.2, 874.4, 874.8, 875.0, 876.0, 877.0, 878.2, 878.4, 878.6, 878.8, 879.2, 879.4, 879.6, 879.8, 911.0, 911.8, 919.0, 919.8, 922.xx, 924.8, 924.9	4	626	515	5.97	82.27	8	1032	805	9.34	78.00
Dislocation	839.xx		9	8	0.09	88.89		176	39	0.45	22.16
Sprains and strains	846.xx-847.xx, 848.3-848.9	6	350	224	2.60	64.00	11	851	399	4.63	46.89
Internal injury	860.xx-869.xx		92	47	0.55	51.09		153	97	1.13	63.40
Injury to blood vessels/nerve, Crushing injury	902.8x, 926.xx, 952.xx-954.xx	4	19	14	0.16	73.68	8	71	50	0.58	70.42
**Upper extremity**											
Fracture	810.xx-819.xx, 829.xx	3	892	750	8.70	84.08		1357	1031	11.96	75.98
Open wound	880.xx-884.xx (excluding xxx.1x)	5	295	211	2.45	71.53	9	400	290	3.36	72.50
Superficial injury	912.0, 912.8, 913.0, 913.8, 914.0, 914.8, 915.0, 915.8	3	129	116	1.35	89.92	7	195	173	2.01	88.72
Contusion	923.xx, 924.8, 924.9	5	622	487	5.65	78.30		1010	762	8.84	75.45
Dislocation	831.xx-834.xx, 839.8, 839.9	3	84	68	0.80	82.14	9	171	122	1.42	71.35
Sprains and strains							12	711	347	4.03	48.81

© 2019 Min L et al. *JAMA Network Open.*

Shoulder and upper arm	840.xx	6	121	74	0.86	61.16					
Elbow and forearm	841.xx	3	15	13	0.15	86.67					
Wrist	842.0x	6	81	50	0.58	61.73					
Hand	842.1x		31	18	0.21	58.07					
Unspecified site of sprain and strain	848.9	3	2	2	0.02	100.00					
Injury to blood vessels/nerve, Crushing injury	903.xx, 927.xx, 955.xx	6	9	3	0.03	33.33	12	26	13	0.15	50.00
**Lower extremity**											
Fracture	820.xx-829.xx	2	1280	1127	13.08	88.05	10	2063	1481	17.18	71.79
Open wound											
Hip and thigh	890.0, 890.2		7	3	0.03	42.86	12	19	10	0.12	52.63
Knee, leg and ankle	891.0, 891.2	5	68	53	0.61	77.94	10	109	81	0.94	74.31
Foot	892.0, 892.2		14	7	0.08	50.00	12	54	24	0.28	44.44
Toe(s)	893.0, 893.2		17	5	0.06	29.41		35	12	0.14	34.29
Multiple and unspecified open wound of lower limb	894.0, 894.2		4	1	0.01	25.00		9	4	0.05	44.44
Superficial injury	916.0, 916.8, 917.0, 917.8	2	103	88	1.02	85.44	7	154	130	1.51	84.42
Contusion	924.xx		862	700	8.12	81.21	10	1474	1048	12.16	71.10
Dislocation			43	26	0.30	60.47					
Hip	835.xx						10	33	24	0.28	72.73
Knee	836.xx						12	478	121	1.40	25.31
Ankle	837.xx						7	12	11	0.13	91.67

© 2019 Min L et al. *JAMA Network Open.*

Foot	838.xx						12	35	16	0.19	45.71
Dislocation of multiple and ill-defined sites	839.8, 839.9						7	5	4	0.05	80.00
Sprains	843.xx-845.xx, 848.9		672	355	4.12	52.83	12	1177	561	6.51	47.66
Injury to blood vessels/nerve, Crushing injury	904.xx, 928.xx, 956.xx	2	7	6	0.07	85.71		25	14	0.16	56.00

Technical notes on grouping International Classification of Disease-9 (ICD-9) diagnostic codes.

Potential Fall-related Injury types identified by ICD-9 codes were organized into a matrix of anatomic regions and injury types. We conceptualized the composite reference standard (E-code for fall or patient-reported fall injury) as similar to a "gold standard" for testing the matrix categories using traditional diagnostic test properties. Of the test properties, we focused on positive predictive value (PPV), or percent of test alternative injuries validated by the composite reference standard, as our criterion to simplify and prioritize the matrix. The second focus of the project was inclusion, or sensitivity (SEN), or the percent of the composite reference standard captured by the matrix categories. First, we collapsed matrix categories low, medium and high accuracy. Low PPV was roughly determined by <70% (gray), medium PPV was 70-79% (blue), high PPV was >80% (orange). For cells less than 20, we grouped together with other categories in the same anatomic region if within one PPV category higher. We grouped the injuries separately using these criteria for acute care (emergency department/hospital/post-hospital nursing home) injuries vs all injuries (acute care plus the outpatient clinic data). Consolidated groups are labeled with group ID number and color coded (orange=high; blue=medium; gray=low). Groups in gray without a group ID number (<50% accuracy) were further refined by specific diagnoses.

© 2019 Min L et al. *JAMA Network Open.*

For example, knee and ankle dislocations were not included in any final category and hip dislocation was moved to lower limb injuries (category 4) based on PPV. Final consolidated categories are listed in Appendix B2.

© 2019 Min L et al. *JAMA Network Open.*

eTable 3. Final Consolidated Injury Groups

Level of Healthcare Acuity	Consolidated Group	Anatomical parts and injury types	ICD 9 codes	Number of observations	Test +	True+	PPV
**ED/hospital / NH only**	**Head injury**	Open wound, superficial injury and contusion of head and face	870.xx-872.6x (excluding xxx.1, 871.5, 871.6), 872.8, 873.0, 873.2x, 873.4x, 873.6x, 873.73, 873.8, 910.0, 910.8, 918.0, 918.9, 920.xx-921.xx, 924.8, 924.9	1383	1602	1378	86.0%
		Fracture of skull and intracranial injury	800.xx-804.xx, 829.xx, 850.xx-854.xx	393			
		Sprains and strains of head and face	848.0, 848.1, 848.2, 848.8, 848.9	9			
		Injury to blood vessels/nerve, crushing injury of head and face	900.xx, 925.xx, 950.xx, 951.xx	2			
		Dislocation of jaw	830.xx, 839.8, 839.9	1			
	**Lower limb fracture and other injury**	Fracture of lower limb	820.xx-829.xx	1280	2080	1759	84.6%
		Contusion of lower limb	924.xx	862			
		Superficial injury of lower limb	916.0, 916.8, 917.0, 917.8	103			
		Injury to blood vessels/nerve, crushing injury of lower limb	904.xx 928.xx, 956.xx	7			
	**Upper limb fracture and other injury**	Fracture of upper limb	810.xx-819.xx, 829.xx	892	1078	912	84.6%
		Superficial injury of upper limb	912.0, 912.8, 913.0, 913.8, 914.0, 914.8, 915.0, 915.8	129			
		Dislocation of upper limb	831.xx-834.xx, 839.8, 839.9	84			
		Sprains and strains of elbow, forearm and unspecified site	841.xx, 848.9	17			

© 2019 Min L et al. *JAMA Network Open.*

	**Neck /trunk fracture and other injury**	Fracture of neck and trunk	805.xx-806.xx, 807.0x-807.1x, 807.2-807.6, 808.xx, 809.xx, 829.xx	798	1344	1075	80.0%
		Open wound, superficial injury and contusion of neck and trunk	874.0x, 874.2, 874.4, 874.8, 875.0, 876.0, 877.0, 878.2, 878.4, 878.6, 878.8, 879.2, 879.4, 879.6, 879.8, 911.0, 911.8, 919.0, 919.8, 922.xx, 924.8, 924.9	626			
		Injury to blood vessels/nerve, crushing injury of neck and trunk	902.8x, 926.xx, 952.xx-954.xx	19			
		Dislocation of neck and trunk	839.xx	9			
	**Upper limb contusion and open wound**	Contusion of upper limb	923.xx, 924.8, 924.9	622	912	689	75.5%
		Open wound of upper limb	880.xx-884.xx (excluding xxx.1x)	295			
		Open wound of knee, leg and ankle	891.0, 891.2	68			
	**Neck/trunk/ upper limb sprains**	Sprains and strains of neck and trunk	846.xx-847.xx, 848.3-848.9	350	658	396	60.2%
		Sprains and strains of shoulder, upper arm, wrist and hand	840.xx, 842.0x, 842.1x	233			
		Internal injury of neck and trunk	860.xx-869.xx	92			
		Injury to blood vessels/nerves and crushing injury of upper limb	903.xx, 927.xx, 955.xx	9			
**All injuries (ED/Hospital/NH and Ambulatory)**	**Head injury and upper/ lower limb superficial injury**	Open wound, Superficial injury, contusion of head and face	870.xx-872.6x (excluding xxx.1, 871.5, 871.6), 872.8, 873.0, 873.2x, 873.4x, 873.6x, 873.73, 873.8, 910.0, 910.8, 918.0, 918.9, 920.xx-921.xx, 924.8, 924.9	1755	2332	1952	83.7%

© 2019 Min L et al. *JAMA Network Open.*

		Fracture of skull and intracranial injury	800.xx-804.xx, 829.xx, 850.xx-854.xx	776			
		Superficial injury of upper limb	912.0, 912.8, 913.0, 913.8, 914.0, 914.8, 915.0, 915.8	195			
		Superficial injury of lower limb	916.0, 916.8, 917.0, 917.8	154			
		Injury to blood vessels/nerve, crushing injury of head and face	900.xx, 925.xx, 950.xx, 951.xx	22			
		Sprains and strains of head and face	848.0, 848.1, 848.2, 848.8, 848.9	21			
		Dislocation of ankle, multiple and ill-defined sites	837.xx, 839.8, 839.9	17			
		Dislocation of jaw	830.xx, 839.8, 839.9	3			
	**Neck/trunk open wound, superficial injury and contusion**	Open wound, superficial injury and contusion of neck and trunk	874.0x, 874.2, 874.4, 874.8, 875.0, 876.0, 877.0, 878.2, 878.4, 878.6, 878.8, 879.2, 879.4, 879.6, 879.8, 911.0, 911.8, 919.0, 919.8, 922.xx, 924.8, 924.9	1032	1092	846	77.5%
		Injury to blood vessels/nerves, crushing injury of neck and trunk	902.8x, 926.xx, 952.xx-954.xx	71			
	**Upper limb fracture and other injury**	Fracture of upper limb	810.xx-819.xx, 829.xx	1357	2253	1679	74.5%
		Contusion of upper limb	923.xx, 924.8, 924.9	1010			
		Open wound of upper limb	880.xx-884.xx (excluding xxx.1x)	400			
		Dislocation of upper limb	831.xx-834.xx, 839.8, 839.9	171			
	**Lower limb fracture and other injury**	Fracture of lower limb	820.xx-829.xx	2063	3255	2290	70.4%
		Contusion of lower limb	924.xx	1474			
		Open wound of knee, leg and ankle	891.0, 891.2	109			
		Dislocation of hip	835.xx	33			

© 2019 Min L et al. *JAMA Network Open.*

	**Neck/trunk fracture and other injury**	Fracture of neck and trunk	805.xx-806.xx, 807.0x-807.1x, 807.2-807.6, 808.xx, 809.xx, 829.xx	1454	2389	1380	57.8%
		Sprains and strains of neck and trunk	846.xx-847.xx, 848.3 - 848.9	851			
		Dislocation of neck and trunk	839.xx	176			
		Internal injury of thorax, abdomen, and pelvis	860.xx-869.xx	153			
	**Upper and lower limb sprains and other injuries unlikely to be due to a fall**	Sprains and strains of lower limb	843.xx-845.xx, 848.9	1177	2405	1027	42.7%
		Sprains and strains of upper limb	840.xx, 841.xx, 842.0x, 842.1x, 848.9	711			
		Dislocation of knee and foot	836.xx, 838.xx	513			
		Open wound of hip, thigh, foot, toe(s) and multiple and unspecified sites of lower limb	890.0, 890.2, 892.0, 892.2, 893.0, 893.2, 894.0, 894.2	117			
		Injury to blood vessels/nerves, crushing injury of upper limb	903.xx, 927.xx, 955.xx	26			
		Injury to blood vessels/nerves, crushing injury of lower limb	904.xx, 928.xx, 956.xx	25			

© 2019 Min L et al. *JAMA Network Open.*

eTable 4. Coding Appendices Overview

**<u>General Overview</u>**<br>• eTable 5 is the required first step for all three algorithms.<br>• If the Acute Care algorithm is desired, then please refer to eTable 5 then 6<br>• If the Balanced algorithm is desired, please refer to eTable 5 then 7<br>• If the Inclusive algorithm is desired, please refer to eTable 5 then 8
**eTable 5: SAS code to analyze Medicare claims eligible for potential fall injury**<br>• Concatenate all the data sources from Medicare inpatient, outpatient (this includes all outpatient clinic and emergency room data) nursing home claims files.<br>• Include all injuries with potential for fall injury (all injuries in ICD-9 800-999 except those types listed below)<br>• Group the injuries into anatomic locations (e.g., head, limb) and type of injury (e.g., fracture, contusion)<br>• Apply inclusions for outpatient clinic data which require confirmation with CPT codes (procedures and imaging) that are specific to anatomic locations (e.g., an arm cast for arm fracture or arm x-ray for arm contusion)<br>• Exclude if E-codes other than fall are found in the claim (e.g., auto accident E-code would exclude a hip fracture claim).<br>• Exclude if advanced cancer or bone metastatic disease is found in the claim
**eTable 6: SAS code for the fall algorithm 1 (Acute care)**<br>• Create episodes of care for all claims within the same category of injury within 180 days of each other.<br>• Create episodes of care by fall-related E codes within 180 days of each other<br>• Exclude any episode of either type that begins with a non-fall E-code<br>• Flag episodes with any E-code for falls plus the 5 consolidated categories of ICD-9 FRI episodes in the Acute Care algorithm. All of these episodes include at least some care in the hospital or emergency room.<br>• The first date of service for each episode is considered to be the date of the fall injury
**eTable 7: SAS code for the fall algorithm 2 (Balanced)**<br>• Create episodes of care for all claims within the same category of injury within 180 days of each other.<br>• Create episodes of care by fall-related E codes within 180 days of each other<br>• Exclude any episode of either type that begins with a non-fall E-code<br>• Flag episodes with any E-code for falls plus the 5 consolidated categories of ICD-9 FRI episodes in the Acute Care algorithm, plus 4 consolidated categories of outpatient clinic FRI episodes that results in the Balanced Algorithm. The first date of service for each episode is considered to be the date of the fall injury
**eTable 8. SAS code for fall algorithm 3 (Inclusive Algorithm)**<br>• Create episodes of care for all claims within the same category of injury within 180 days of each other.<br>• Create episodes of care by fall-related E codes within 180 days of each other<br>• Exclude any episode of either type that begins with a non-fall E-code<br>• Flag episodes with any E-code for falls, the 5 consolidated categories of ICD-9 FRI episodes in the Acute Care algorithm, plus 4 consolidated categories of outpatient

© 2019 Min L et al. *JAMA Network Open.*

clinic FRI episodes in the Balanced Algorithm, plus 2 consolidated categories of outpatient clinic FRI episodes to form the Inclusive Algorithm. The first date of service for each episode is considered to be the date of the fall injury
**Technical notes on potential fall-related injury diagnosis codes**<br><br>In eTable 5, when we collected all claims with injuries that could plausibly be caused by a fall, we considered all International Classification of Diseases-9 injury codes from 800 to 999. Of these, we excluded these types of injuries considered to be highly implausible to be due to a fall:<br>• Burns, all types<br>• Electric injury<br>• Stings<br>• Bites<br>• Foreign objects<br>• Punctures deep into the pleura, peritoneum, or deep hollow structure<br>• Traumatic amputations<br>• Contusions or lacerations of deeper hollow organs (esophageal, bowel, bladder, vagina)<br>• Late-effects or subsequent follow-up care for prior injury
**Technical notes on E-codes**<br><br>E-codes identify low-velocity falls including ground-level falls, falls from a chair, commode, bed, or fewer than one flight of stairs (e-codes). We excluded high-velocity falls such as those from a second-floor balcony or involving sports activities, as they are not likely due to frailty and gait impairment. If a fall E-code was co-coded with a non-fall mechanism (e.g., a car versus pedestrian accident where the provider also submitted an e-code for the pedestrian's fall) then the event was not considered as a fall.

eTable 5. SAS Code to Analyze Medicare Claims Eligible for Potential Fall Injury

```
/* Fill in the LIBNAME statements below with the location of the datasets */
libname fall "location of datasets";

/* Step 1. Define all potential fall-related claims (ICD 9 diagnosis code) *******************/
data Fall_event;
  set fall.Claims;          * Create a concatenated data set combining all claims, labeling the claim source
                                 as Inpatient (IP), Skilled nursing facilities (SN),
                                 Outpatient (OP) and Denominator (PB) files;

 cms + 1;

 /* Define the ED visits in the outpatient file using revenue codes */
array RVCNTR {45} RVCNTR01 - RVCNTR45;
        do i = 1 to 45;
          if OP = 1 & (RVCNTR{i} in ("0450", "0451", "0452", "0453", "0454", "0455",
                                             "0456", "0457", "0458", "0459", "0981")) then OP_ED = 1;
        end;

/* Define places of care (hospital, emergency, ambulatory = outpatient clinic, and nursing home)*/
 Hospital = 0; if IP = 1 then Hospital = 1;
 Emergency = 0; if OP_ED = 1 then Emergency = 1;
 Ambulatory = 0; if (OP = 1 & OP_ED ne 1) or PB = 1 then Ambulatory = 1;
 NursingHome = 0; if SN = 1 then NursingHome = 1;

 /* Define matrix of categories of fall-related claims (ICD 9 code) based on anatomy and injury type*/
 ICD9_1f = 0; ICD9_1c = 0; ICD9_1d = 0; ICD9_1s = 0; ICD9_1o = 0;

 ICD9_2f = 0; ICD9_2f_rib = 0; ICD9_2f_pelvis = 0; ICD9_2f_other = 0;
 ICD9_2c = 0; ICD9_2d = 0; ICD9_2s = 0; ICD9_2i = 0; ICD9_2o = 0;

 ICD9_3f = 0; ICD9_3f_clavicle = 0; ICD9_3f_humerus = 0; ICD9_3f_radius = 0; ICD9_3f_navicular = 0;
 ICD9_3f_hand_metacarpal = 0; ICD9_3f_hand_phalanges = 0; ICD9_3f_hand_multi = 0; ICD9_3f_other = 0;
```

© 2019 Min L et al. *JAMA Network Open.*

```
ICD9_3c_open = 0; ICD9_3c_superficial = 0; ICD9_3c_contusion = 0;
ICD9_3d = 0; ICD9_3d_shoulder = 0; ICD9_3d_elbow = 0; ICD9_3d_wrist = 0; ICD9_3d_other = 0;
ICD9_3s = 0; ICD9_3s_shoulder = 0; ICD9_3s_elbow = 0; ICD9_3s_wrist = 0; ICD9_3s_hand = 0; ICD9_3s_unspecified = 0;
ICD9_3o = 0;

ICD9_4f = 0; ICD9_4f_hip = 0; ICD9_4f_femur = 0; ICD9_4f_patella = 0; ICD9_4f_ankle = 0; ICD9_4f_other = 0;
ICD9_4c_open_hip = 0; ICD9_4c_open_knee = 0; ICD9_4c_open_foot = 0; ICD9_4c_open_toe = 0; ICD9_4c_open_multi = 0;
ICD9_4c_superficial = 0; ICD9_4c_contusion = 0;
ICD9_4d = 0; ICD9_4d_hip = 0; ICD9_4d_knee = 0; ICD9_4d_ankle = 0; ICD9_4d_foot = 0; ICD9_4d_multi = 0;
ICD9_4s = 0; ICD9_4o = 0;


array a{13} i0 i1 - i12;        *Categorize Claim Diagnosis Code PDGNS_CD, DGNSCD01 - DGNSCD12
                                      to numeric values: i0, i1 - i12;
        do i = 1 to 13;

        /* Head and face */
        if              800 <= a{i} < 805 or 829 <= a{i} < 830 or 850 <= a{i} < 855
                        then ICD9_1f = 1;                         *Fracture of skull and head trauma;
        if              (870 <= a{i} < 872.7 and a{i} not in (870.1, 871.1, 871.5, 871.6, 872.1)) or
                        a{i} in (872.8, 873.0, 873.8, 873.73) or 873.2 <= a{i} < 873.3 or
                        873.4 <= a{i} < 873.5 or 873.6 <= a{i} < 873.7 or
                        a{i} in (910, 910.8, 918, 918.9) or 920 <= a{i} < 922 or a{i} in (924.8, 924.9)
                        then ICD9_1c = 1;                     *Open wound, Superficial injury, Contusion;
        if              830 <= a{i} < 831 or a{i} in (839.8, 839.9)
                        then ICD9_1d = 1;                         *Dislocation of jaw;
        if              a{i} in (848.0, 848.1, 848.2, 848.8, 848.9)
                        then ICD9_1s = 1;                         *Sprains and strains;
        if              900 <= a{i} < 901 or 925 <= a{i} < 926 or 950 <= a{i} < 951 or 951 <= a{i} < 952
                        then ICD9_1o = 1;                         *Injury to blood vessels/nerve, Crushing injury;
        ICD9_1 = 1*(sum(ICD9_1f, ICD9_1c, ICD9_1d, ICD9_1s, ICD9_1o) >= 1);

        /* Neck and trunk */
```

© 2019 Min L et al. *JAMA Network Open.*

```
if              807 <= a{i} < 807.2 then ICD9_2f_rib = 1;            *Fracture of rib(s);
else if  808 <= a{i} < 809      then ICD9_2f_pelvis = 1;      *Fracture of pelvis;
else if  805 <= a{i} < 807 or a{i} in (807.2, 807.3, 807.4, 807.5, 807.6) or
                809 <= a{i} < 810 or 829 <= a{i} < 830
                then ICD9_2f_other = 1;                                   *Fracture of others;
                ICD9_2f = 1*(sum(ICD9_2f_rib, ICD9_2f_pelvis, ICD9_2f_other) >= 1); *Fracture;
if              874 <= a{i} < 874.1 or
                a{i} in (874.2, 874.4, 874.8, 875.0, 876.0, 877.0,
                        878.2, 878.4, 878.6, 878.8, 879.2, 879.4, 879.6, 879.8) or
        a{i} in (911.0, 911.8, 919.0, 919.8) or 922 <= a{i} < 923 or a{i} in (924.8, 924.9)
                then ICD9_2c = 1;                                         *Open wound, Superficial injury, Contusion;
if              839 <= a{i} < 839.6 or 839.6 <= a{i} < 840
                then ICD9_2d = 1;                                         *Dislocation;
if              846 <= a{i} < 848 or 848.3 <= a{i} < 848.6 or a{i} in (848.8, 848.9)
                then ICD9_2s = 1;                                         *Sprains and strains;
if              860 <= a{i} < 870
                then ICD9_2i = 1;                                         *Internal injury;
if              902.8 <= a{i} < 903 or 926 <= a{i} < 927 or 952 <= a{i} < 955
                then ICD9_2o = 1;                                         *Injury to blood vessels/nerve, Crushing injury;
ICD9_2 = 1*(sum(ICD9_2f, ICD9_2c, ICD9_2d, ICD9_2s, ICD9_2i, ICD9_2o) >= 1);

/* Upper extremity */
if              810 <= a{i} < 811 then ICD9_3f_clavicle = 1;          *Fracture of clavicle;
else if  812 <= a{i} < 813 then ICD9_3f_humerus = 1;       *Fracture of humerus;
else if  813 <= a{i} < 814 then ICD9_3f_radius = 1;*Fracture of radius and ulna;
else if  a{i} in (814, 814.01, 814.09, 814.1, 814.11, 814.19)
                then ICD9_3f_navicular = 1;                        *Fracture of carpal bone(s);
else if  815 <= a{i} < 816 then ICD9_3f_hand_metacarpal = 1;      *Fracture of metacarpal bone(s);
else if  816 <= a{i} < 817 then ICD9_3f_hand_phalanges = 1;       *Fracture of one or more phalanges of hand;
else if  817 <= a{i} < 818 then ICD9_3f_hand_multi = 1;           *Multiple fractures of hand bones;
else if  811 <= a{i} < 812 or 814.02 <= a{i} < 814.09 or 814.12 <= a{i} < 814.19 or
                818 <= a{i} < 820 or 829 <= a{i} < 830 then ICD9_3f_other = 1;   *Fracture of others;
                ICD9_3f = 1*(sum(ICD9_3f_clavicle, ICD9_3f_humerus, ICD9_3f_radius,
```

© 2019 Min L et al. *JAMA Network Open.*

```
                              ICD9_3f_navicular, ICD9_3f_hand_metacarpal, ICD9_3f_hand_phalanges,
                              ICD9_3f_hand_multi, ICD9_3f_other) >= 1);        *Fracture;
if          (880 <= a{i} < 885 and
     a{i} not in (880.10, 880.11, 880.12, 880.13, 880.19, 881.10, 881.11, 881.12, 882.1, 883.1, 884.1))
            then ICD9_3c_open = 1;                                  *Open wound;
if          a{i} in (912.0, 912.8, 913.0, 913.8, 914.0, 914.8, 915.0, 915.8)
            then ICD9_3c_superficial = 1;                           *Superficial injury;
if          923 <= a{i} < 924 or a{i} in (924.8, 924.9)
            then ICD9_3c_contusion = 1;                             *Contusion;
if          831 <= a{i} < 832 then ICD9_3d_shoulder = 1;       *Dislocation of shoulder;
else if  832 <= a{i} < 833 then ICD9_3d_elbow = 1;             *Dislocation of elbow;
else if  833 <= a{i} < 834 then ICD9_3d_wrist = 1;        *Dislocation of wrist;
else if  834 <= a{i} < 835 or 839.8 <= a{i} < 840 then ICD9_3d_other = 1;      *Dislocation of others;
            ICD9_3d = 1*(sum(ICD9_3d_shoulder, ICD9_3d_elbow,
                              ICD9_3d_wrist, ICD9_3d_other) >= 1);  *Dislocation;
if          840 <= a{i} < 841 then ICD9_3s_shoulder = 1;       *Sprains and strains of shoulder and upper arm;
else if  841 <= a{i} < 842 then ICD9_3s_elbow = 1;        *Sprains and strains of elbow and forearm;
else if  842 <= a{i} < 842.1 then ICD9_3s_wrist = 1;      *Sprains and strains of wrist;
else if  842.1 <= a{i} < 842.2 then ICD9_3s_hand = 1;     *Sprains and strains of hand;
else if  a{i} in (848.9) then ICD9_3s_unspecified = 1;    *Unspecified site of sprain and strain;
            ICD9_3s = 1*(sum(ICD9_3s_shoulder, ICD9_3s_elbow, ICD9_3s_wrist,
                              ICD9_3s_hand, ICD9_3s_unspecified) >= 1);     *Sprains and strains;
if          903 <= a{i} < 904 or 927 <= a{i} < 928 or 955 <= a{i} < 956
            then ICD9_3o = 1;                                       *Injury to blood vessels/nerve, Crushing injury;
ICD9_3 = 1*(sum(ICD9_3f, ICD9_3c_open, ICD9_3c_superficial, ICD9_3c_contusion,
                        ICD9_3d, ICD9_3s, ICD9_3o) >= 1);

/* Lower extremity */
if          820 <= a{i} < 821 then ICD9_4f_hip = 1;            *Fracture of neck of femur;
else if  821 <= a{i} < 822 or 823 <= a{i} < 824
            then ICD9_4f_femur = 1;      *Fracture of other and unspecified parts of femur or tibia and fibula;
else if  822 <= a{i} < 823 then ICD9_4f_patella = 1;      *Fracture of patella;
else if  824 <= a{i} < 825 then ICD9_4f_ankle = 1;        *Fracture of ankle;
```

© 2019 Min L et al. *JAMA Network Open.*

```
else if  825 <= a{i} < 830 then ICD9_4f_other = 1;             *Fracture of other;
                    ICD9_4f = 1*(sum(ICD9_4f_hip, ICD9_4f_femur, ICD9_4f_patella,
                                        ICD9_4f_ankle, ICD9_4f_other) >= 1);   *Fracture;
if                  890 <= a{i} < 891 and a{i} ne 890.1 then ICD9_4c_open_hip = 1;  *Open wound of hip and thigh;
else if  891 <= a{i} < 892 and a{i} ne 891.1 then ICD9_4c_open_knee = 1; *Open wound of knee, leg and ankle;
else if  892 <= a{i} < 893 and a{i} ne 892.1 then ICD9_4c_open_foot = 1; *Open wound of foot;
else if  893 <= a{i} < 894 and a{i} ne 893.1 then ICD9_4c_open_toe = 1;  *Open wound of toe(s);
else if  894 <= a{i} < 895 and a{i} ne 894.1
                    then ICD9_4c_open_multi = 1;                            *Multiple and unspecified open wound of lower limb;
if                  a{i} in (916.0, 916.8, 917.0, 917.8)
                    then ICD9_4c_superficial = 1;                           *Superficial injury;
if                  924 <= a{i} < 924.6 or a{i} in (924.8, 924.9)
                    then ICD9_4c_contusion = 1;                             *Contusion;
if                  835 <= a{i} < 836 then ICD9_4d_hip = 1;             *Dislocation of hip;
else if  836 <= a{i} < 837 then ICD9_4d_knee = 1;             *Dislocation of knee;
else if  837 <= a{i} < 838 then ICD9_4d_ankle = 1;            *Dislocation of ankle;
else if 838 <= a{i} < 839 then ICD9_4d_foot = 1;              *Dislocation of foot;
else if 839.8 <= a{i} < 840 then ICD9_4d_multi = 1;                 *Dislocation of multiple and ill-defined sites;
                    ICD9_4d = 1*(sum(ICD9_4d_hip, ICD9_4d_knee, ICD9_4d_ankle,
                       ICD9_4d_foot, ICD9_4d_multi) >= 1);   *Dislocation;
if                  843 <= a{i} < 846 or a{i} in (848.9)
                    then ICD9_4s = 1;                                       *Sprains;
if                  904 <= a{i} < 905 or 928 <= a{i} < 929 or 956 <= a{i} < 957
                    then ICD9_4o = 1;                                       *Injury to blood vessels/nerve, Crushing injury;
ICD9_4 = 1*(sum(ICD9_4f, ICD9_4c_open_hip, ICD9_4c_open_knee, ICD9_4c_open_foot, ICD9_4c_open_toe,
                      ICD9_4c_open_multi, ICD9_4c_superficial, ICD9_4c_contusion, ICD9_4d, ICD9_4s, ICD9_4o) >= 1);

ICD9 = sum(of ICD9_1 - ICD9_4);
fall_ICD9 = 1*(ICD9 >= 1);

/* label for Stage 4 cancer or Metastatic bone disease */
if         170 <= a{i} < 171 or 196 <= a{i} < 199 or a{i} = 733.1 then cancer = 1;
```

© 2019 Min L et al. *JAMA Network Open.*

```
        end;

array ecode{14} ei0 ei1 - ei12 e;      *Convert claims with E Code DGNS_E into numeric value e, and captured additional
                                                 E codes from Claim Diagnosis Code PDGNS_CD, DGNSCD01 - DGNSCD12;
        do i = 1 to 14;

                if 800 <= ecode{i} < 849 or 890 <= ecode{i} < 900 then motor_e = 1;
                if 880 <= ecode{i} < 881 or ecode{i} in (884.0, 884.2, 884.3, 884.4, 884.5, 884.6, 884.9) or
          ecode{i} = 885.9 or 887 <= ecode{i} < 889 then do;
                        fall_e = 1;
                fall_e880 = 1*(880 <= ecode{i} < 881);
                        fall_e884 = 1*(884 <= ecode{i} < 885);
                        fall_e885 = 1*(885 <= ecode{i} < 886);
                        fall_e887 = 1*(887 <= ecode{i} < 888);
                        fall_e888 = 1*(888 <= ecode{i} < 889);
                                                        end;
                if motor_e = . & fall_e = . & ecode{i} ne . then other_e = 1;
                if 849 <= ecode{i} < 850 then other_e = .;

        end;

/* Define Cause Of Injury (COI): 1 = Motor vehicles accidents; 2 = Others; 3 = Falls */
if motor_e ne . then COI = 1;
else if other_e ne . and fall_e = . then COI = 2;
else if fall_e ne . then COI = 3;
else if motor_e = . & fall_e = . & other_e = . then COI = .;

/* Define falls injury related procedure code */
%let cast = "29000","29001","29002","29003","29004","29005","29006","29007","29008","29009","29010","29011",
"29012","29013","29014","29015","29016","29017","29018","29019","29020","29021","29022","29023","29024","29025",
"29026","29027","29028","29029","29030","29031","29032","29033","29034","29035","29036","29037","29038","29039",
"29040","29041","29042","29043","29044","29045","29046","29047","29048","29049","29050","29051","29052","29053",
"29054","29055","29056","29057","29058","29059","29060","29061","29062","29063","29064","29065","29066","29067",
```

© 2019 Min L et al. *JAMA Network Open.*

```
"29068","29069","29070","29071","29072","29073","29074","29075","29076","29077","29078","29079","29080","29081",
"29082","29083","29084","29085","29086","29305","29306","29307","29308","29309","29310","29311","29312","29313",
"29314","29315","29316","29317","29318","29319","29320","29321","29322","29323","29324","29325","29326","29327",
"29328","29329","29330","29331","29332","29333","29334","29335","29336","29337","29338","29339","29340","29341",
"29342","29343","29344","29345","29346","29347","29348","29349","29350","29351","29352","29353","29354","29355",
"29356","29357","29358","29359","29360","29361","29362","29363","29364","29365","29366","29367","29368","29369",
"29370","29371","29372","29373","29374","29375","29376","29377","29378","29379","29380","29381","29382","29383",
"29384","29385","29386","29387","29388","29389","29390","29391","29392","29393","29394","29395","29396","29397",
"29398","29399","29400","29401","29402","29403","29404","29405","29406","29407","29408","29409","29410","29411",
"29412","29413","29414","29415","29416","29417","29418","29419","29420","29421","29422","29423","29424","29425",
"29426","29427","29428","29429","29430","29431","29432","29433","29434","29435","29436","29437","29438","29439",
"29440","29441","29442","29443","29444","29445","29446","29447","29448","29449","29450";
%let splint = "29105","29106","29107","29108","29109","29110","29111","29112","29113","29114","29115","29116",
"29117","29118","29119","29120","29121","29122","29123","29124","29125","29126","29127","29128","29129","29130",
"29131","29505","29506","29507","29508","29509","29510","29511","29512","29513","29514","29515";
%let repair_hip ="27230","27231","27232","27233","27234","27235","27236","27237","27238","27239","27240","27241",
"27242","27243","27244","27245","27246","27247","27248";
%let repair_pelvis = "27193","27194","27215","27216","27217","27218","27219","27220","27221","27222","27223",
"27224","27225","27226","27227","27228";
%let repair_rib = "21800","21805","21810";
%let repair_clavicle = "23500","23501","23502","23503","23504","23505","23506","23507","23508","23509","23510",
"23511","23512","23513","23514","23515";
%let repair_humerus = "23600","23601","23602","23603","23604","23605","23606","23607","23608","23609","23610",
"23611","23612","23613","23614","23615","23616","23617","23618","23619","23620","23621","23622","23623","23624",
"23625","23626","23627","23628","23629","23630","23665","23666","23667","23668","23669","23670","23671","23672",
"23673","23674","23675","23676","23677","23678","23679","23680","24500","24501","24502","24503","24504","24505",
"24506","24507","24508","24509","24510","24511","24512","24513","24514","24515","24516","24517","24518","24519",
"24520","24521","24522","24523","24524","24525","24526","24527","24528","24529","24530","24531","24532","24533",
"24534","24535","24536","24537","24538","24539","24540","24541","24542","24543","24544","24545","24546","24547",
"24548","24549","24550","24551","24552","24553","24554","24555","24556","24557","24558","24559","24560","24561",
"24562","24563","24564","24565","24566","24567","24568","24569","24570","24571","24572","24573","24574","24575",
"24576","24577","24578","24579","24580","24581","24582","24583","24584","24585","24586","24587";
%let repair_radius = "24586","24587","24620","24635","24650","24651","24652","24653","24654","24655","24656",
```

© 2019 Min L et al. *JAMA Network Open.*

```
"24657","24658","24659","24660","24661","24662","24663","24664","24665","24666","24667","24668","24669","24670",
"24671","24672","24673","24674","24675","24676","24677","24678","24679","24680","24681","24682","24683","24684",
"24685","25500","25501","25502","25503","25504","25505","25506","25507","25508","25509","25510","25511","25512",
"25513","25514","25515","25516","25517","25518","25519","25520","25521","25522","25523","25524","25525","25526",
"25527","25528","25529","25530","25531","25532","25533","25534","25535","25536","25537","25538","25539","25540",
"25541","25542","25543","25544","25545","25546","25547","25548","25549","25550","25551","25552","25553","25554",
"25555","25556","25557","25558","25559","25560","25561","25562","25563","25564","25565","25566","25567","25568",
"25569","25570","25571","25572","25573","25574","25575","25576","25577","25578","25579","25580","25581","25582",
"25583","25584","25585","25586","25587","25588","25589","25590","25591","25592","25593","25594","25595","25596",
"25597","25598","25599","25600","25601","25602","25603","25604","25605","25606","25607","25608","25609","25611",
"25620","25650","25651","25652";
%let repair_navicular = "25622","25623","25624","25625","25626","25627","25628";
%let repair_hand_metacarpal = "26600","26605","26607","26608","26615","26645","26650","26665","26740","26746";
%let repair_hand_phalanges = "26720","26725","26727","26735","26740","26742","26746","26750","26755","26756","26765";
%let repair_femur = "27500","27501","27502","27503","27504","27505","27506","27507","27508","27509","27510",
"27511","27512","27513","27514","27530","27531","27532","27533","27534","27535","27536","27537","27538","27539",
"27540","27750","27751","27752","27753","27754","27755","27756","27757","27758","27759","27780","27781","27782",
"27783","27784","27785","27786","27787","27788","27789","27790","27791","27792","27824","27825","27826","27827","27828";
%let repair_patella = "27520","27524";
%let repair_ankle = "27760","27761","27762","27763","27764","27765","27766","27767","27768","27769","27808",
"27809","27810","27811","27812","27813","27814","27815","27816","27817","27818","27819","27820","27821","27822",
"27823","28430","28431","28432","28433","28434","28435","28436","28437","28438","28439","28440","28441","28442",
"28443","28444","28445";
%let repair_shoulder = "23650","23655","23660";
%let repair_elbow = "24600","24605","24615";
%let repair_wrist = "25660","25670","25671","25675","25676","25690","25695";
%let repair_knee = "27550","27552","27556","27557","27558","27560","27562","27566";
%let image_rib = "71010","71015","71020","71021","71022","71023","71030","71034","71035","71100","71101","71110",
"71111","71250","71260","71270","71275","71550","71551","71552","71553","71554","71555";
%let image_clavicle = "71010","71015","71020","71021","71022","71023","71030","71034","71035","71250","71260",
"71270","71275","71550","71551","71552","71553","71554","71555","73000","73020","73030","73040";
%let image_humerus = "73020","73030","73040","73060","73200","73201","73202","73206","73218","73219","73220","73225";
%let image_hand_metacarpal = "73120","73121","73122","73123","73124","73125","73126","73127","73128","73129",
```

© 2019 Min L et al. *JAMA Network Open.*

```
"73130","73131","73132","73133","73134","73135","73136","73137","73138","73139","73140","73141","73142","73143",
"73144","73145","73146","73147","73148","73149","73150","73151","73152","73153","73154","73155","73156","73157",
"73158","73159","73160","73161","73162","73163","73164","73165","73166","73167","73168","73169","73170","73171",
"73172","73173","73174","73175","73176","73177","73178","73179","73180","73181","73182","73183","73184","73185",
"73186","73187","73188","73189","73190","73191","73192","73193","73194","73195","73196","73197","73198","73199",
"73200","73201","73202","73203","73204","73205","73206","73207","73208","73209","73210","73211","73212","73213",
"73214","73215","73216","73217","73218","73219","73220","73221","73222","73223","73224","73225";
%let image_hand_phalanges = "73120","73121","73122","73123","73124","73125","73126","73127","73128","73129",
"73130","73131","73132","73133","73134","73135","73136","73137","73138","73139","73140","73141","73142","73143",
"73144","73145","73146","73147","73148","73149","73150","73151","73152","73153","73154","73155","73156","73157",
"73158","73159","73160","73161","73162","73163","73164","73165","73166","73167","73168","73169","73170","73171",
"73172","73173","73174","73175","73176","73177","73178","73179","73180","73181","73182","73183","73184","73185",
"73186","73187","73188","73189","73190","73191","73192","73193","73194","73195","73196","73197","73198","73199",
"73200","73201","73202","73203","73204","73205","73206","73207","73208","73209","73210","73211","73212","73213",
"73214","73215","73216","73217","73218","73219","73220","73221","73222","73223","73224","73225";
%let image_hand_multi_f = "73120","73121","73122","73123","73124","73125","73126","73127","73128","73129",
"73130","73131","73132","73133","73134","73135","73136","73137","73138","73139","73140","73141","73142","73143",
"73144","73145","73146","73147","73148","73149","73150","73151","73152","73153","73154","73155","73156","73157",
"73158","73159","73160","73161","73162","73163","73164","73165","73166","73167","73168","73169","73170","73171",
"73172","73173","73174","73175","73176","73177","73178","73179","73180","73181","73182","73183","73184","73185",
"73186","73187","73188","73189","73190","73191","73192","73193","73194","73195","73196","73197","73198","73199",
"73200","73201","73202","73203","73204","73205","73206","73207","73208","73209","73210","73211","73212","73213",
"73214","73215","73216","73217","73218","73219","73220","73221","73222","73223","73224","73225";
%let image_shoulder = "71010","71015","71020","71021","71022","71023","71030","71034","71035","71250","71260",
"71270","71275","71550","71551","71552","71553","71554","71555","73020","73030","73040";
%let image_elbow = "73070","73080","73085","73200","73201","73202","73203","73204","73205","73206","73221",
"73222","73223","73224","73225";
%let image_wrist = "73100","73101","73102","73103","73104","73105","73106","73107","73108","73109","73110",
"73111","73112","73113","73114","73115","73200","73201","73202","73203","73204","73205","73206","73221","73222",
"73223","73224","73225";
%let image_knee = "73560","73561","73562","73563","73564","73565","73566","73567","73568","73569","73570",
"73571","73572","73573","73574","73575","73576","73577","73578","73579","73580","73700","73701","73702","73703",
"73704","73705","73706","73721","73722","73723","73724","73725";
```

© 2019 Min L et al. *JAMA Network Open.*

```
%let Min_head_face = "12013","12032","13121","70100","70110","70120","70130","70134","70140","70150","70160",
"70190","70200","70250","70260","70450","70486";
%let Min_neck_trunk = "12032","13121","27197","71100","71101","71110","71111","71120","71130","72010","72020",
"72040","72050","72070","72072","72074","72080","72100","72110","72114","72125","72131","72170","72190","72192",
"72220","73010","73050","74174";
%let Min_upper_extremity = "12032","13121","23570","73000","73020","73030","73060","73070","73080","73090",
"73100","73110","73120","73130","73140";
%let Min_lower_extremity = "12032","13121","27130","27702","72200","72202","73500","73502","73510","73520","73550","73560",
"73562","73564","73565","73590","73600","73610","73620","73630","73650","73660","73700";

array cpt {45} HCPSCD01 - HCPSCD45;
        if Emergency = 1 or Ambulatory = 1 then
        do i = 1 to 45;

          if cpt{i} in (&Min_head_face.) then do;
                CPT_1f = 1; CPT_1c = 1; CPT_1d = 1; CPT_1s = 1; CPT_1o = 1; end;

          if cpt{i} in (&repair_rib.) or cpt{i} in (&image_rib.)  then CPT_2f_rib = 1;
          if cpt{i} in (&repair_pelvis.)  then CPT_2f_pelvis = 1;
          if cpt{i} in (&Min_neck_trunk.) then do;
                CPT_2f_rib = 1; CPT_2f_pelvis = 1; CPT_2f_other = 1;
        CPT_2c = 1; CPT_2d = 1; CPT_2s = 1; CPT_2i = 1; CPT_2o = 1; end;

          if cpt{i} in (&repair_clavicle.) or cpt{i} in (&image_clavicle.) then CPT_3f_clavicle = 1;
          if cpt{i} in (&repair_humerus.) or cpt{i} in (&splint.) or cpt{i} in (&image_humerus.)
                then CPT_3f_humerus = 1;
          if cpt{i} in (&repair_radius.) or cpt{i} in (&cast.) or cpt{i} in (&splint.) then CPT_3f_radius = 1;
          if cpt{i} in (&repair_navicular.) or cpt{i} in (&cast.) or cpt{i} in (&splint.) then CPT_3f_navicular = 1;
          if cpt{i} in (&repair_hand_metacarpal.) or cpt{i} in (&splint.) or cpt{i} in (&image_hand_metacarpal.)
                then CPT_3f_hand_metacarpal = 1;
          if cpt{i} in (&repair_hand_phalanges.) or cpt{i} in (&splint.) or cpt{i} in (&image_hand_phalanges.)
                then CPT_3f_hand_phalanges = 1;
          if CPT_3f_hand_metacarpal = 1 or CPT_3f_hand_phalanges = 1 then CPT_3f_hand_multi = 1;
```

© 2019 Min L et al. *JAMA Network Open.*

```
if cpt{i} in (&repair_shoulder.) or cpt{i} in (&splint.) or cpt{i} in (&image_shoulder.)
        then CPT_3d_shoulder = 1;
    if cpt{i} in (&repair_elbow.) or cpt{i} in (&splint.) or cpt{i} in (&image_elbow.) then CPT_3d_elbow = 1;
    if cpt{i} in (&repair_wrist.) or cpt{i} in (&cast.) or cpt{i} in (&splint.) or cpt{i} in (&image_wrist.)
        then CPT_3d_wrist = 1;
    if cpt{i} in (&Min_upper_extremity.) then do;
                CPT_3f_clavicle = 1; CPT_3f_humerus = 1; CPT_3f_radius = 1; CPT_3f_navicular = 1;
                CPT_3f_hand_metacarpal = 1; CPT_3f_hand_phalanges = 1; CPT_3f_hand_multi = 1; CPT_3f_other = 1;
                CPT_3c_open = 1; CPT_3c_superficial = 1; CPT_3c_contusion = 1;
                CPT_3d_shoulder = 1; CPT_3d_elbow = 1; CPT_3d_wrist = 1; CPT_3d_other = 1;
                CPT_3s_shoulder = 1; CPT_3s_elbow = 1; CPT_3s_wrist = 1; CPT_3s_hand = 1; CPT_3s_unspecified = 1;
                CPT_3o = 1;   end;

    if cpt{i} in (&repair_hip.) then CPT_4f_hip = 1;
    if cpt{i} in (&repair_femur.) or cpt{i} in (&cast.) or cpt{i} in (&splint.) then CPT_4f_femur = 1;
    if cpt{i} in (&repair_patella.) or cpt{i} in (&splint.) then CPT_4f_patella = 1;
    if cpt{i} in (&repair_ankle.) then CPT_4f_ankle = 1;
    if cpt{i} in (&repair_knee.) or cpt{i} in (&cast.) or cpt{i} in (&splint.) or cpt{i} in (&image_knee.)
        then CPT_4d_knee = 1;
    if cpt{i} in (&Min_lower_extremity.) then do;
        CPT_4f_hip = 1; CPT_4f_femur = 1; CPT_4f_patella = 1; CPT_4f_ankle = 1; CPT_4f_other = 1;
        CPT_4c_open_hip = 1; CPT_4c_open_knee = 1; CPT_4c_open_foot = 1; CPT_4c_open_toe = 1;
        CPT_4c_open_multi = 1; CPT_4c_superficial = 1; CPT_4c_contusion = 1; CPT_4d_hip = 1; CPT_4d_knee = 1;
        CPT_4d_ankle = 1; CPT_4d_foot = 1; CPT_4d_multi = 1; CPT_4s = 1; CPT_4o = 1; end;

   end;

drop i;
run;

/* Step 2. If the ICD-fall-related diagnosis claim from Step 1 is associated with
      stage 3 cancer or metastatic bone disease, then exclude as a fall-related claim***************************/
data Check(drop = cancer) noCheck Cancer;
```

© 2019 Min L et al. *JAMA Network Open.*

```
 set Fall_event;
 if COI = . and fall_ICD9 = 1 then output Check;
 else output noCheck;

 if cancer = 1 then output Cancer;
run;
proc sql;
 create table Check_cancer
 as select a.*, b.cancer
 from Check as a
 left join Cancer as b on (a.hhidpn = b.hhidpn and ((a.From_DT - 30 <= b.Thru_DT and b.Thru_DT <= a.FROM_DT) or
                                                    (a.Thru_DT <= b.From_DT and b.From_DT <= a.Thru_DT + 30)));
quit;
proc sql;
 create table Check_cancer1
 as select cms, 1*(sum(cancer) >= 1) as fall_cancer
 from Check_cancer
 group by 1
 order by 1;
quit;
proc sort data = Check; by cms;
data Check1;
 merge Check Check_cancer1;
 by cms;
run;

data fall.fall_event;
 set Check1 noCheck;
 fall_event = fall_ICD9;
 if fall_cancer = 1 then fall_event = 0;
run;
proc datasets library = work nolist;
 delete Fall_event Check noCheck Cancer Check_cancer Check_cancer1 Check1;
```

© 2019 Min L et al. *JAMA Network Open.*

```
run;

/* Step 3. Confirm fall-related claims from outpatient clinic or ER that have no E codes using CPT codes *************/
data CPT noCPT;
 set fall.fall_event;
 if (Emergency = 1 or Ambulatory = 1) & fall_event = 1 & COI = . then output CPT;
 else output noCPT;
run;

data noCPT;
 set noCPT;
 %let vlist1 = 1f 1c 1d 1s 1o
             2f_rib 2f_pelvis 2f_other 2c 2d 2s 2i 2o
             3f_clavicle 3f_humerus 3f_radius 3f_navicular 3f_hand_metacarpal 3f_hand_phalanges
                     3f_hand_multi 3f_other 3c_open 3c_superficial 3c_contusion 3d_shoulder 3d_elbow 3d_wrist 3d_other
                     3s_shoulder 3s_elbow 3s_wrist 3s_hand 3s_unspecified 3o
                     4f_hip 4f_femur 4f_patella 4f_ankle 4f_other 4c_open_hip 4c_open_knee 4c_open_foot
                     4c_open_toe 4c_open_multi 4c_superficial 4c_contusion 4d_hip 4d_knee 4d_ankle 4d_foot 4d_multi 4s 4o;
 %let nwords = %sysfunc(countw(&vlist1));
 %macro loop;
 %do i = 1 %to &nwords;
 %let a = %scan(&vlist1, &i);
 ICD9_&a._cpt = ICD9_&a;
 %end;
 %mend;
 %loop;
 ICD9_2f_cpt = ICD9_2f;
 ICD9_3f_cpt = ICD9_3f; ICD9_3d_cpt = ICD9_3d; ICD9_3s_cpt = ICD9_3s;
 ICD9_4f_cpt = ICD9_4f; ICD9_4d_cpt = ICD9_4d;
 fall_event_cpt = fall_event;
run;

data Dx; run;
```

© 2019 Min L et al. *JAMA Network Open.*

```
 %macro within10days;
 %do i = 1 %to &nwords;
 %let code = %scan(&vlist1, &i);
 proc sql;
 create table Dx_&code
 as select a.HHIDPN, a.FROM_DT, a.cms, a.ICD9_&code,
           b.FROM_DT as CPT_FROM_DT, b.CPT_&code
 from CPT as a
 left join CPT as b on (a.hhidpn = b.hhidpn and a.FROM_DT - 10 <= b.FROM_DT and b.FROM_DT <= a.FROM_DT + 10)
 where a.ICD9_&code = 1 and b.CPT_&code = 1;
quit;
data Dx;
 set Dx Dx_&code;
proc datasets library = work nolist; delete Dx_&code;
run;
 %end;
 %mend;
%within10days;
proc sort data = Dx; by cms; run;

proc sql;
 create table CPT1
      as select cms,

                    (sum(ICD9_1f) >= 1) as ICD9_1f_cpt,
                    (sum(ICD9_1c) >= 1) as ICD9_1c_cpt,
                    (sum(ICD9_1d) >= 1) as ICD9_1d_cpt,
                    (sum(ICD9_1s) >= 1) as ICD9_1s_cpt,
                    (sum(ICD9_1o) >= 1) as ICD9_1o_cpt,

                    (sum(ICD9_2f_rib) >= 1) as ICD9_2f_rib_cpt,
                    (sum(ICD9_2f_pelvis) >= 1) as ICD9_2f_pelvis_cpt,
                    (sum(ICD9_2f_other) >= 1) as ICD9_2f_other_cpt,
```

© 2019 Min L et al. *JAMA Network Open.*

```
(sum(ICD9_2c) >= 1) as ICD9_2c_cpt,
(sum(ICD9_2d) >= 1) as ICD9_2d_cpt,
(sum(ICD9_2s) >= 1) as ICD9_2s_cpt,
(sum(ICD9_2i) >= 1) as ICD9_2i_cpt,
(sum(ICD9_2o) >= 1) as ICD9_2o_cpt,

(sum(ICD9_3f_clavicle) >= 1) as ICD9_3f_clavicle_cpt,
(sum(ICD9_3f_humerus) >= 1) as ICD9_3f_humerus_cpt,
(sum(ICD9_3f_radius) >= 1) as ICD9_3f_radius_cpt,
(sum(ICD9_3f_navicular) >= 1) as ICD9_3f_navicular_cpt,
(sum(ICD9_3f_hand_metacarpal) >= 1) as ICD9_3f_hand_metacarpal_cpt,
(sum(ICD9_3f_hand_phalanges) >= 1) as ICD9_3f_hand_phalanges_cpt,
(sum(ICD9_3f_hand_multi) >= 1) as ICD9_3f_hand_multi_cpt,
(sum(ICD9_3f_other) >= 1) as ICD9_3f_other_cpt,
(sum(ICD9_3c_open) >= 1) as ICD9_3c_open_cpt,
(sum(ICD9_3c_superficial) >= 1) as ICD9_3c_superficial_cpt,
(sum(ICD9_3c_contusion) >= 1) as ICD9_3c_contusion_cpt,
(sum(ICD9_3d_shoulder) >= 1) as ICD9_3d_shoulder_cpt,
(sum(ICD9_3d_elbow) >= 1) as ICD9_3d_elbow_cpt,
(sum(ICD9_3d_wrist) >= 1) as ICD9_3d_wrist_cpt,
(sum(ICD9_3d_other) >= 1) as ICD9_3d_other_cpt,
(sum(ICD9_3s_shoulder) >= 1) as ICD9_3s_shoulder_cpt,
(sum(ICD9_3s_elbow) >= 1) as ICD9_3s_elbow_cpt,
(sum(ICD9_3s_wrist) >= 1) as ICD9_3s_wrist_cpt,
(sum(ICD9_3s_hand) >= 1) as ICD9_3s_hand_cpt,
(sum(ICD9_3s_unspecified) >= 1) as ICD9_3s_unspecified_cpt,
(sum(ICD9_3o) >= 1) as ICD9_3o_cpt,

(sum(ICD9_4f_hip) >= 1) as ICD9_4f_hip_cpt,
(sum(ICD9_4f_femur) >= 1) as ICD9_4f_femur_cpt,
(sum(ICD9_4f_patella) >= 1) as ICD9_4f_patella_cpt,
(sum(ICD9_4f_ankle) >= 1) as ICD9_4f_ankle_cpt,
(sum(ICD9_4f_other) >= 1) as ICD9_4f_other_cpt,
```

© 2019 Min L et al. *JAMA Network Open.*

```
                    (sum(ICD9_4c_open_hip) >= 1) as ICD9_4c_open_hip_cpt,
                    (sum(ICD9_4c_open_knee) >= 1) as ICD9_4c_open_knee_cpt,
                    (sum(ICD9_4c_open_foot) >= 1) as ICD9_4c_open_foot_cpt,
                    (sum(ICD9_4c_open_toe) >= 1) as ICD9_4c_open_toe_cpt,
                    (sum(ICD9_4c_open_multi) >= 1) as ICD9_4c_open_multi_cpt,
                    (sum(ICD9_4c_superficial) >= 1) as ICD9_4c_superficial_cpt,
                    (sum(ICD9_4c_contusion) >= 1) as ICD9_4c_contusion_cpt,

                    (sum(ICD9_4d_hip) >= 1) as ICD9_4d_hip_cpt,
                    (sum(ICD9_4d_knee) >= 1) as ICD9_4d_knee_cpt,
                    (sum(ICD9_4d_ankle) >= 1) as ICD9_4d_ankle_cpt,
                    (sum(ICD9_4d_foot) >= 1) as ICD9_4d_foot_cpt,
                    (sum(ICD9_4d_multi) >= 1) as ICD9_4d_multi_cpt,
                    (sum(ICD9_4s) >= 1) as ICD9_4s_cpt,
                    (sum(ICD9_4o) >= 1) as ICD9_4o_cpt
 from Dx
 where cms ne .
 group by 1;
quit;
data CPT2;
 merge CPT CPT1;
 by cms;
 ICD9_2f_cpt = 1*(sum(ICD9_2f_rib_cpt, ICD9_2f_pelvis_cpt, ICD9_2f_other_cpt) >= 1);
 ICD9_3f_cpt = 1*(sum(ICD9_3f_clavicle_cpt, ICD9_3f_humerus_cpt, ICD9_3f_radius_cpt, ICD9_3f_navicular_cpt,
                      ICD9_3f_hand_metacarpal_cpt, ICD9_3f_hand_phalanges_cpt, ICD9_3f_hand_multi_cpt, ICD9_3f_other_cpt) >=
                      1);
 ICD9_3d_cpt = 1*(sum(ICD9_3d_shoulder_cpt, ICD9_3d_elbow_cpt, ICD9_3d_wrist_cpt, ICD9_3d_other_cpt) >= 1);
 ICD9_3s_cpt = 1*(sum(ICD9_3s_shoulder_cpt, ICD9_3s_elbow_cpt, ICD9_3s_wrist_cpt, ICD9_3s_hand_cpt,
                    ICD9_3s_unspecified_cpt) >= 1);
 ICD9_4f_cpt = 1*(sum(ICD9_4f_hip_cpt, ICD9_4f_femur_cpt, ICD9_4f_patella_cpt, ICD9_4f_ankle_cpt,
                    ICD9_4f_other_cpt) >= 1);
 ICD9_4d_cpt = 1*(sum(ICD9_4d_hip_cpt, ICD9_4d_knee_cpt, ICD9_4d_ankle_cpt, ICD9_4d_foot_cpt, ICD9_4d_multi_cpt) >= 1);
 fall_event_cpt = (sum(ICD9_1f_cpt, ICD9_1c_cpt, ICD9_1d_cpt, ICD9_1s_cpt, ICD9_1o_cpt,
```

© 2019 Min L et al. *JAMA Network Open.*

```
                    ICD9_2f_cpt, ICD9_2c_cpt, ICD9_2d_cpt, ICD9_2s_cpt, ICD9_2i_cpt, ICD9_2o_cpt,
                    ICD9_3f_cpt, ICD9_3c_open_cpt, ICD9_3c_superficial_cpt, ICD9_3c_contusion_cpt, ICD9_3d_cpt,
                              ICD9_3s_cpt, ICD9_3o_cpt,
                    ICD9_4f_cpt, ICD9_4c_open_hip_cpt, ICD9_4c_open_knee_cpt, ICD9_4c_open_foot_cpt,
                              ICD9_4c_open_toe_cpt, ICD9_4c_open_multi_cpt, ICD9_4c_superficial_cpt, ICD9_4c_contusion_cpt,
                              ICD9_4d_cpt, ICD9_4s_cpt, ICD9_4o_cpt) >= 1);
run;

data fall.fall_event_cpt;
 set noCPT CPT2;
 proc sort;
 by hhidpn From_DT Thru_DT cms;
run;

proc datasets library = work nolist;
 delete NoCPT CPT CPT1 CPT2 CPT3 Dx;
run;
```

© 2019 Min L et al. *JAMA Network Open.*

eTable 6. SAS Code for the Fall Algorithm 1 (Acute Care)

```
/* Fill in the LIBNAME statements below with the location of the outputs */
libname Alg1 "location of outputs";
/* Fill in the %INCLUDE statements below to bring the "fall claims.sas" program */
%Include "location of program\fall claims.sas";

/* Step 4 Define episodes of care (claims in the same injury category, <180 days)*******************/
data Falls0;
 set fall.fall_event_cpt;
 if fall_event_cpt = 1 or COI = 3;
 proc sort; by hhidpn From_DT Thru_DT;
run;
%macro fall_epi;
data fall_event;
 set Falls0;
 if &fall_event_condi;

 by hhidpn;
 format lag_Thru_DT date9.;
 lag_Thru_DT = lag(Thru_DT);
 if first.hhidpn then lag_Thru_DT = .;
 l = From_DT - lag_Thru_DT;

 if l ne . & l > 180 then id + 1;
 if first.hhidpn then id = 0;

 where &POC;
run;

proc sql;
 create table &fall_event._epi
 as select hhidpn, id,
                min(From_DT) as Start_DT format Date9.,
```

© 2019 Min L et al. *JAMA Network Open.*

```
                max(Thru_DT) as End_DT format Date9.,
                sum(&fall_event) as N_claims,
                1*(sum(&fall_event) >= 1) as fall_episode
 from fall_event
 group by 1, 2;
quit;

data &fall_event._epi;
 set &fall_event._epi;
 format episode_id $15.;
 episode_id = "&a";
run;
%mend;

/* Step 4.1 Identify the episodes with fall-related E codes *******************************************************/
%let POC = Hospital = 1 or NursingHome = 1 or Emergency = 1 or Ambulatory = 1;
%let fall_event_condi = COI = 3 and fall_e880 = 1; %let fall_event = fall_e880; %let a = e880; %fall_epi;
%let fall_event_condi = COI = 3 and fall_e884 = 1; %let fall_event = fall_e884; %let a = e884; %fall_epi;
%let fall_event_condi = COI = 3 and fall_e885 = 1; %let fall_event = fall_e885; %let a = e885; %fall_epi;
%let fall_event_condi = COI = 3 and fall_e887 = 1; %let fall_event = fall_e887; %let a = e887; %fall_epi;
%let fall_event_condi = COI = 3 and fall_e888 = 1; %let fall_event = fall_e888; %let a = e888; %fall_epi;
data E_episode;
 set Fall_e880_epi Fall_e884_epi Fall_e885_epi Fall_e887_epi Fall_e888_epi;
 drop fall_episode;
 where fall_episode = 1;
proc sort; by hhidpn Start_DT End_DT;
run;

/* Step 4.2 Identify the episodes with ICD 9 codes in the Acute Care Algorithm****************************************/
%let vlist2 = 1f 1c 1d 1s 1o
                   2f 2c 2d 2s 2i 2o
          3f 3c_open 3c_superficial 3c_contusion 3d
                   3s 3s_shoulder 3s_elbow 3s_wrist 3s_hand 3s_unspecified 3o
```

© 2019 Min L et al. *JAMA Network Open.*

```
                    4f 4c_open_hip 4c_open_knee 4c_open_foot 4c_open_toe 4c_open_multi
                    4c_superficial 4c_contusion 4d 4d_hip 4d_knee 4d_ankle 4d_foot 4d_multi 4s 4o;
%macro ICD9_epi;
data ICD9_epi; run;
%let nwords = %sysfunc(countw(&vlist2));

%do i = 1 %to &nwords;
%let a = %scan(&vlist2, &i);
%let fall_event_condi = fall_event_cpt = 1 and ICD9_&a._cpt = 1;
%let fall_event = ICD9_&a._cpt;
%fall_epi;
data ICD9_epi;
set ICD9_epi &fall_event._epi;
if hhidpn ne '';
run;
proc datasets library = work nolist; delete &fall_event._epi; run;
%end;
proc sort data = ICD9_epi; by hhidpn Start_DT End_DT; run;
%mend;
%let POC = Hospital = 1 or NursingHome = 1 or Emergency = 1;
%ICD9_epi;

/* Step 4.2.1 Disqualify any episode that begin with a non-fall E-code ********************************************/
data NoFalls0;
 set fall.fall_event_cpt;
 if fall_event_cpt = 1 and COI in (1,2) and (Hospital = 1 or NursingHome = 1 or Emergency = 1);
run;
%macro NoFalls;
data NoFalls; run;
%let nwords = %sysfunc(countw(&vlist2));

%do i = 1 %to &nwords;
%let a = %scan(&vlist2, &i);
```

© 2019 Min L et al. *JAMA Network Open.*

```
data NoFalls1;
 set NoFalls0;
 if ICD9_&a._cpt = 1;
 format id $15.;
 id = "&a";
 keep hhidpn From_DT COI id;
run;
data NoFalls;
 set NoFalls NoFalls1;
run;
%end;
data NoFalls; set NoFalls; where hhidpn ne ''; run;
%mend NoFalls;
%NoFalls;
proc sql;
 create table ICD9_episode
 as select a.hhidpn, a.id, a.Start_DT, a.End_DT, a.N_claims, a.episode_id
 from ICD9_epi as a
 left join NoFalls as b on (a.hhidpn = b.hhidpn and a.Start_DT = b.From_DT and a.episode_id = b.id)
 where a.episode_id in ('1f', '1c', '1d', '1s', '1o',
                                   '2f', '2c', '2d', '2o',
                        '3f', '3c_open', '3c_superficial', '3c_contusion', '3d', '3s_elbow', '3s_unspecified',
                                   '4f', '4c_open_knee', '4c_superficial', '4c_contusion', '4d', '4o') and b.COI = .;
quit;

/* Alg1.Fall_episode is a dataset with all fall-related episodes of care based on the Acute Care Algorithm */
data Alg1.Fall_episode;
 set E_episode ICD9_episode;
drop id;
label hhidpn = 'PatientID'
    Start_DT = 'First date of episode'
        End_DT = 'Last date of episode'
        N_claims = 'Number of claims'
```

© 2019 Min L et al. *JAMA Network Open.*

```
        episode_id = 'Anatomical parts and injury types';
proc sort; by hhidpn Start_DT End_DT episode_id;
run;

proc datasets library = work nolist;
 delete Fall_e880_epi Fall_e884_epi Fall_e885_epi Fall_e887_epi Fall_e888_epi E_episode
         Falls0 Fall_event NoFalls NoFalls0 NoFalls1 ICD9_epi ICD9_episode;
run;
```

© 2019 Min L et al. *JAMA Network Open.*

eTable 7. SAS Code for the Fall Algorithm 2 (Balanced)

```
/* Fill in the LIBNAME statements below with the location of the outputs */
libname Alg2 ”location of outputs”;
/* Fill in the %INCLUDE statements below to bring the “fall claims.sas” program */
%Include ”location of program\fall claims.sas”;

/* Step 4 Define potential fall-related episodes of care**********************************************/
data Falls0;
 set fall.fall_event_cpt;
 if fall_event_cpt = 1 or COI = 3;
 proc sort; by hhidpn From_DT Thru_DT;
run;
%macro fall_epi;
data fall_event;
 set Falls0;
 if &fall_event_condi;

 by hhidpn;
 format lag_Thru_DT date9.;
 lag_Thru_DT = lag(Thru_DT);
 if first.hhidpn then lag_Thru_DT = .;
 l = From_DT - lag_Thru_DT;

 if l ne . & l > 180 then id + 1;
 if first.hhidpn then id = 0;

 where &POC;
run;

proc sql;
 create table &fall_event._epi
 as select hhidpn, id,
                min(From_DT) as Start_DT format Date9.,
```

© 2019 Min L et al. *JAMA Network Open.*

```
                max(Thru_DT) as End_DT format Date9.,
                sum(&fall_event) as N_claims,
                1*(sum(&fall_event) >= 1) as fall_episode
 from fall_event
 group by 1, 2;
quit;

data &fall_event._epi;
 set &fall_event._epi;
 format episode_id $15.;
 episode_id = "&a";
run;
%mend;

/* Step 4.1 Identify the episodes of care with an E-code for fall as the mechanism of injury
*****************************************************************************/
%let POC = Hospital = 1 or NursingHome = 1 or Emergency = 1 or Ambulatory = 1;
%let fall_event_condi = COI = 3 and fall_e880 = 1; %let fall_event = fall_e880; %let a = e880; %fall_epi;
%let fall_event_condi = COI = 3 and fall_e884 = 1; %let fall_event = fall_e884; %let a = e884; %fall_epi;
%let fall_event_condi = COI = 3 and fall_e885 = 1; %let fall_event = fall_e885; %let a = e885; %fall_epi;
%let fall_event_condi = COI = 3 and fall_e887 = 1; %let fall_event = fall_e887; %let a = e887; %fall_epi;
%let fall_event_condi = COI = 3 and fall_e888 = 1; %let fall_event = fall_e888; %let a = e888; %fall_epi;
data E_episode;
 set Fall_e880_epi Fall_e884_epi Fall_e885_epi Fall_e887_epi Fall_e888_epi;
 drop fall_episode;
 where fall_episode = 1;
proc sort; by hhidpn Start_DT End_DT;
run;

/* Step 4.2 Identify the episodes of care by categories (anatomic region and type of injury)
*************************************************************************/
%let vlist2 = 1f 1c 1d 1s 1o
                         2f 2c 2d 2s 2i 2o
```

© 2019 Min L et al. *JAMA Network Open.*

```
            3f 3c_open 3c_superficial 3c_contusion 3d 3s_shoulder 3s_elbow 3s_wrist 3s_hand 3s_unspecified 3o
                      4f 4c_open_hip 4c_open_knee 4c_open_foot 4c_open_toe 4c_open_multi
                 4c_superficial 4c_contusion 4d 4s 4o;
%macro ICD9_epi(level);
data ICD9_epi&level; run;
%let nwords = %sysfunc(countw(&vlist2));

%do i = 1 %to &nwords;
%let a = %scan(&vlist2, &i);
%let fall_event_condi = fall_event_cpt = 1 and ICD9_&a._cpt = 1;
%let fall_event = ICD9_&a._cpt;
%fall_epi;
data ICD9_epi&level;
set ICD9_epi&level &fall_event._epi;
if hhidpn ne '';
run;
proc datasets library = work nolist; delete &fall_event._epi; run;
%end;
proc sort data = ICD9_epi&level; by hhidpn Start_DT End_DT; run;
%mend;
%let POC = Hospital = 1 or NursingHome = 1 or Emergency = 1;
%ICD9_epi(12);

%let POC = Hospital = 1 or NursingHome = 1 or Emergency = 1 or Ambulatory = 1;
%ICD9_epi(123);

/* Step 4.2.1 Disqualify any episode that begins with a non-fall E-code ***********************************************/
data NoFalls0_12 NoFalls0_123;
 set fall.fall_event_cpt;
 if fall_event_cpt = 1 and COI in (1,2) and (Hospital = 1 or NursingHome = 1 or Emergency = 1) then output NoFalls0_12;
 if fall_event_cpt = 1 and COI in (1,2) then output NoFalls0_123;
run;
%macro NoFalls(level);
```

© 2019 Min L et al. *JAMA Network Open.*

```
data NoFalls&level; run;
%let nwords = %sysfunc(countw(&vlist2));

%do i = 1 %to &nwords;
%let a = %scan(&vlist2, &i);
data NoFalls;
 set NoFalls0_&level;
 if ICD9_&a._cpt = 1;
 format id $15.;
 id = "&a";
 keep hhidpn From_DT COI id;
run;
data NoFalls&level;
 set NoFalls&level NoFalls;
run;
%end;
data NoFalls&level; set NoFalls&level; where hhidpn ne ''; run;
%mend NoFalls;
%NoFalls(12); %NoFalls(123);
proc sql;
 create table ICD9_episode12
 as select a.hhidpn, a.id, a.Start_DT, a.End_DT, a.N_claims, a.episode_id
 from ICD9_epi12 as a
 left join NoFalls12 as b on (a.hhidpn = b.hhidpn and a.Start_DT = b.From_DT and a.episode_id = b.id)
 where a.episode_id in ('2f', '2d', '2s', '2i',
                        '3s_shoulder', '3s_elbow', '3s_wrist', '3s_hand', '3s_unspecified', '3o',
                        '4f', '4c_open_knee', '4c_contusion', '4o') and b.COI = .;
quit;
proc sql;
 create table ICD9_episode123
 as select a.hhidpn, a.id, a.Start_DT, a.End_DT, a.N_claims, a.episode_id
 from ICD9_epi123 as a
 left join NoFalls123 as b on (a.hhidpn = b.hhidpn and a.Start_DT = b.From_DT and a.episode_id = b.id)
```

© 2019 Min L et al. *JAMA Network Open.*

```
 where a.episode_id in ('1f', '1c', '1d', '1s', '1o',
                                    '2c', '2o',
                        '3f', '3c_open', '3c_superficial', '3c_contusion', '3d',
                                            '4c_superficial', '4d_ankle', '4d_multi') and b.COI = .;
quit;
data ICD9_episode;
 set ICD9_episode12 ICD9_episode123;
run;


/* Alg2.Fall_episode is a dataset with fall-related episodes based on Balanced Algorithm */
data Alg2.Fall_episode;
 set E_episode ICD9_episode;
drop id;
label hhidpn = 'PatientID'
      Start_DT = 'First date of episode'
          End_DT = 'Last date of episode'
          N_claims = 'Number of claims'
          episode_id = 'Anatomical parts and injury types';
proc sort; by hhidpn Start_DT End_DT episode_id;
run;

proc datasets library = work nolist;
 delete Fall_e880_epi Fall_e884_epi Fall_e885_epi Fall_e887_epi Fall_e888_epi E_episode
       Falls0 Fall_event NoFalls0_12 NoFalls0_123 NoFalls NoFalls12 NoFalls123 ICD9_epi12 ICD9_epi123 ICD9_episode12
       ICD9_episode123 ICD9_episode;
run;
```

© 2019 Min L et al. *JAMA Network Open.*

eTable 8. SAS Code for Fall Algorithm 3 (Inclusive Algorithm)

```
/* Fill in the LIBNAME statements below with the location of the outputs */
libname Alg3 ”location of outputs”;
/* Fill in the %INCLUDE statements below to bring the “fall claims.sas” program */
%Include ”location of program\fall claims.sas”;

/* Step 4 Define episodes of potentially fall-related care*********************************************************/
data Falls0;
 set fall.fall_event_cpt;
 if fall_event_cpt = 1 or COI = 3;
 proc sort; by hhidpn From_DT Thru_DT;
run;
%macro fall_epi;
data fall_event;
 set Falls0;
 if &fall_event_condi;

 by hhidpn;
 format lag_Thru_DT date9.;
 lag_Thru_DT = lag(Thru_DT);
 if first.hhidpn then lag_Thru_DT = .;
 l = From_DT - lag_Thru_DT;

 if l ne . & l > 180 then id + 1;
 if first.hhidpn then id = 0;

 where &POC;
run;

proc sql;
 create table &fall_event._epi
 as select hhidpn, id,
                min(From_DT) as Start_DT format Date9.,
```

© 2019 Min L et al. *JAMA Network Open.*

```
                max(Thru_DT) as End_DT format Date9.,
                sum(&fall_event) as N_claims,
                1*(sum(&fall_event) >= 1) as fall_episode
 from fall_event
 group by 1, 2;
quit;

data &fall_event._epi;
 set &fall_event._epi;
 format episode_id $15.;
 episode_id = "&a";
run;
%mend;

/* Step 4.1 Identify the episodes with E codes for fall-related mechanism of injury
****************************************************************************/
%let POC = Hospital = 1 or NursingHome = 1 or Emergency = 1 or Ambulatory = 1;
%let fall_event_condi = COI = 3 and fall_e880 = 1; %let fall_event = fall_e880; %let a = e880; %fall_epi;
%let fall_event_condi = COI = 3 and fall_e884 = 1; %let fall_event = fall_e884; %let a = e884; %fall_epi;
%let fall_event_condi = COI = 3 and fall_e885 = 1; %let fall_event = fall_e885; %let a = e885; %fall_epi;
%let fall_event_condi = COI = 3 and fall_e887 = 1; %let fall_event = fall_e887; %let a = e887; %fall_epi;
%let fall_event_condi = COI = 3 and fall_e888 = 1; %let fall_event = fall_e888; %let a = e888; %fall_epi;
data E_episode;
 set Fall_e880_epi Fall_e884_epi Fall_e885_epi Fall_e887_epi Fall_e888_epi;
 drop fall_episode;
 where fall_episode = 1;
proc sort; by hhidpn Start_DT End_DT;
run;

/* Step 4.2 Identify episodes by categories (anatomic region and injury type) based on ICD 9 codes ******************/
%let vlist2 = 1f 1c 1d 1s 1o
                        2f 2c 2d 2s 2i 2o
             3f 3c_open 3c_superficial 3c_contusion 3d 3s_shoulder 3s_elbow 3s_wrist 3s_hand 3s_unspecified 3o
```

© 2019 Min L et al. *JAMA Network Open.*

```
                        4f 4c_open_hip 4c_open_knee 4c_open_foot 4c_open_toe 4c_open_multi 4c_superficial 4c_contusion 4d 4s 4o;
%macro ICD9_epi(level);
data ICD9_epi&level; run;
%let nwords = %sysfunc(countw(&vlist2));

%do i = 1 %to &nwords;
%let a = %scan(&vlist2, &i);
%let fall_event_condi = fall_event_cpt = 1 and ICD9_&a._cpt = 1;
%let fall_event = ICD9_&a._cpt;
%fall_epi;
data ICD9_epi&level;
set ICD9_epi&level &fall_event._epi;
if hhidpn ne '';
run;
proc datasets library = work nolist; delete &fall_event._epi; run;
%end;
proc sort data = ICD9_epi&level; by hhidpn Start_DT End_DT; run;
%mend;
%let POC = Hospital = 1 or NursingHome = 1 or Emergency = 1;
%ICD9_epi(12);

%let POC = Hospital = 1 or NursingHome = 1 or Emergency = 1 or Ambulatory = 1;
%ICD9_epi(123);

/* Step 4.2.1 Disqualify any episode that begins with a non-fall E-code ********************************************/
data NoFalls0_12 NoFalls0_123;
 set fall.fall_event_cpt;
 if fall_event_cpt = 1 and COI in (1,2) and (Hospital = 1 or NursingHome = 1 or Emergency = 1) then output NoFalls0_12;
 if fall_event_cpt = 1 and COI in (1,2) then output NoFalls0_123;
run;
%macro NoFalls(level);
data NoFalls&level; run;
%let nwords = %sysfunc(countw(&vlist2));
```

© 2019 Min L et al. *JAMA Network Open.*

```
%do i = 1 %to &nwords;
%let a = %scan(&vlist2, &i);
data NoFalls;
 set NoFalls0_&level;
 if ICD9_&a._cpt = 1;
 format id $15.;
 id = "&a";
 keep hhidpn From_DT COI id;
run;
data NoFalls&level;
 set NoFalls&level NoFalls;
run;
%end;
data NoFalls&level; set NoFalls&level; where hhidpn ne ''; run;
%mend NoFalls;
%NoFalls(12); %NoFalls(123);
proc sql;
 create table ICD9_episode12
 as select a.hhidpn, a.id, a.Start_DT, a.End_DT, a.N_claims, a.episode_id
 from ICD9_epi12 as a
 left join NoFalls12 as b on (a.hhidpn = b.hhidpn and a.Start_DT = b.From_DT and a.episode_id = b.id)
 where a.episode_id in ('3s_shoulder', '3s_elbow', '3s_wrist', '3s_hand', '3s_unspecified', '3o',
                     '4o') and b.COI = .;
quit;
proc sql;
 create table ICD9_episode123
 as select a.hhidpn, a.id, a.Start_DT, a.End_DT, a.N_claims,
                       a.episode_id
 from ICD9_epi123 as a
 left join NoFalls123 as b on (a.hhidpn = b.hhidpn and a.Start_DT = b.From_DT
                                                                   and a.episode_id = b.id)
 where a.episode_id in ('1f', '1c', '1d', '1s', '1o',
```

© 2019 Min L et al. *JAMA Network Open.*

```
                                    '2f', '2d', '2s', '2i', '2c', '2o',
                     '3f', '3c_open', '3c_superficial', '3c_contusion', '3d',
                                         '4f', '4c_open_knee', '4c_superficial', '4c_contusion', '4d_hip', '4d_ankle', '4d_multi') and
           b.COI = .;
quit;
data ICD9_episode;
 set ICD9_episode12 ICD9_episode123;
run;


/* Alg3.Fall_episode is a dataset with fall-related episodes based on Inclusive Algorithm */
data Alg3.Fall_episode;
 set E_episode ICD9_episode;
drop id;
label hhidpn = 'PatientID'
     Start_DT = 'First date of episode'
          End_DT = 'Last date of episode'
          N_claims = 'Number of claims'
          episode_id = 'Anatomical parts and injury types';
proc sort; by hhidpn Start_DT End_DT episode_id;
run;

proc datasets library = work nolist;
 delete Fall_e880_epi Fall_e884_epi Fall_e885_epi Fall_e887_epi Fall_e888_epi E_episode
             Falls0 Fall_event NoFalls0_12 NoFalls0_123 NoFalls NoFalls12 NoFalls123 ICD9_epi12 ICD9_epi123 ICD9_episode12
             ICD9_episode123 ICD9_episode;
run;
```

© 2019 Min L et al. *JAMA Network Open.*

eTable 9. Sensitivity Analysis for Superficial Injuries

Superficial injury of upper extremity (N = 195)

Additional fracture or dislocation	Additional other minor injuries	Observations with additional injury	Percent validated
0	0	19	84.2%
0	1	84	89.3%
1	0	21	85.7%
1	1	71	90.1%
		195	88.7%

Contusion of upper extremity (N = 1,010)

Additional fracture or dislocation	Additional other minor injuries	Observations with additional injury	Percent validated
0	0	181	48.1%
0	1	404	80.2%
1	0	100	67.0%
1	1	325	87.4%
		1010	75.4%

Superficial injury of lower extremity (N = 154)

Additional fracture or dislocation	Additional other minor injuries	Observations with additional injury	Percent validated
0	0	22	68.2%
0	1	67	83.6%
1	0	11	81.8%
1	1	54	92.6%
		154	84.4%

Contusion of lower extremity (N = 1,474)

Additional fracture or dislocation	Additional other minor injuries	Observations with additional injury	Percent validated
0	0	376	45.2%
0	1	476	77.1%
1	0	184	66.9%
1	1	438	88.6%
		1474	71.1%

We performed this sensitivity analysis in response to higher than expected validity of the superficial injuries and contusions. We test whether validity was substantially inflated by major injuries that were co-existing with the minor injuries. Superficial injury was subcategorized by whether or not there was a co-existing injury within that time period with an additional major or minor injury. We concluded that for superficial injuries of the upper extremity, the validity was uniformly high, regardless of co-existence of other major or minor injuries. For superficial injury of the lower extremity, contusions of the lower extremity and contusions of the upper extremity, validity was increased by the presence of another minor injury more than the presence of a major injury.

© 2019 Min L et al. *JAMA Network Open.*

eTable 10. 3 Algorithm Validity With and Without Using CPT Code Criteria

	Test +		True +	**Sensitivity**	**PPV ***
Using CPT criteria** to confirm outpatient clinic injury episodes					
Fall Algorithm 1	6365	12.7%	5353	62.1%	88.6%
Fall Algorithm 2	7339	14.6%	5629	65.3%	83.2%
Fall Algorithm 3	8777	17.4%	5894	98.4%	75.2%
Without using CPT criteria ** to confirm outpatient clinic injury episodes					
Fall Algorithm 1	6891	13.7%	5449	63.2%	84.7%
Fall Algorithm 2	10261	20.4%	6072	70.4%	66.2%
Fall Algorithm 3	14016	27.9%	6537	75.8%	53.2%

PPV = Positive Predictive Value:

* Using potential test FRIs within 6 months of interviews

** Common Procedural Terminology (CPT) criteria modified from UCLA/RAND[16] method. This method adds CPT code criteria in addition to the diagnostic codes to confirm when considering an eligible fall injury episode from a non-emergency room outpatient clinic claim.

© 2019 Min L et al. *JAMA Network Open.*

eTable 11. Link to Replace *ICD-9* Codes in All 3 Algorithms to *ICD-10*

Please visit https://sites.google.com/umich.edu/minfallalgorithmsignup. The link will ask for an email address that we can use to send updates for any of the SAS codes and the ICD-9 to ICD-10 crosswalk. The ICD-10 crosswalk is still in beta testing, so please contact with questions and suggested updates to lmingroup@umich.edu.

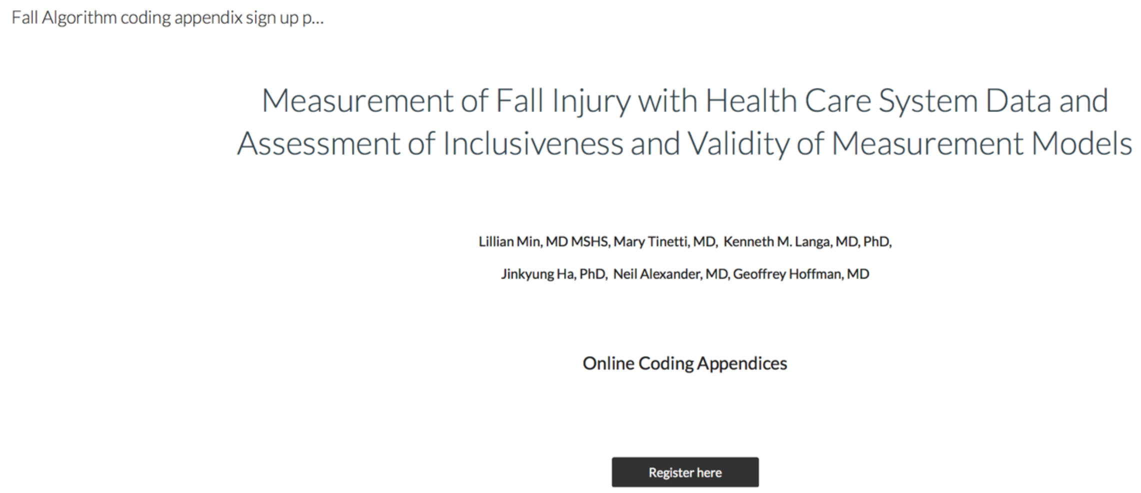

Fall Algorithm coding appendix sign up p...

Measurement of Fall Injury with Health Care System Data and Assessment of Inclusiveness and Validity of Measurement Models

Lillian Min, MD MSHS, Mary Tinetti, MD, Kenneth M. Langa, MD, PhD,
Jinkyung Ha, PhD, Neil Alexander, MD, Geoffrey Hoffman, MD

Online Coding Appendices

Register here

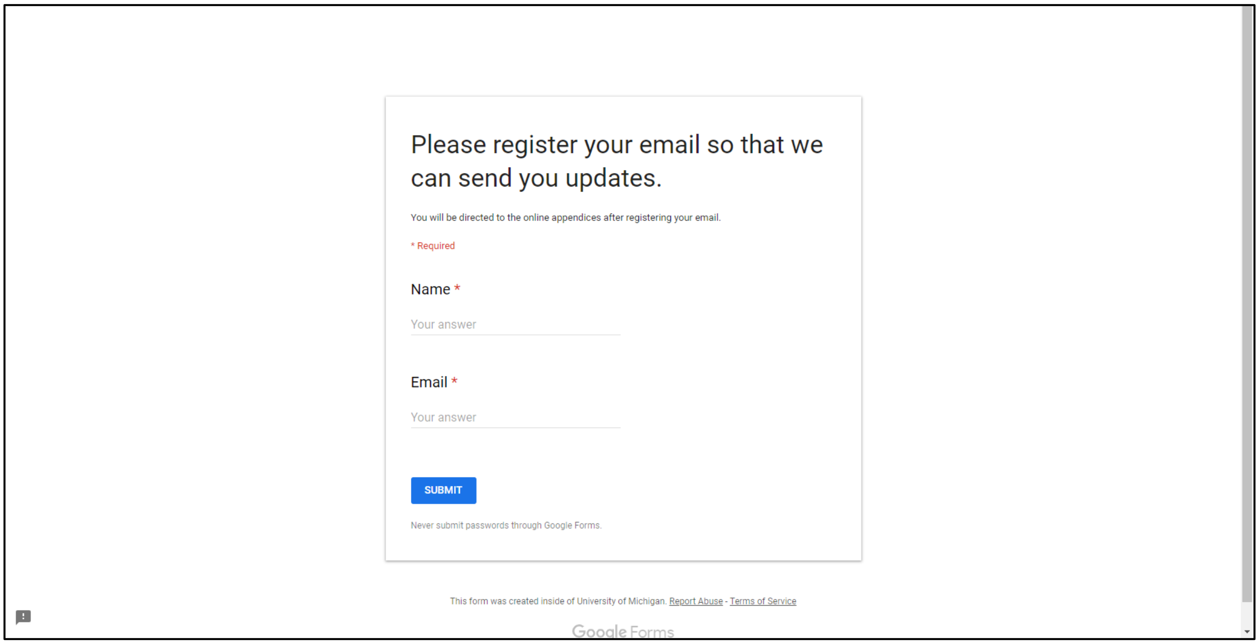

Please register your email so that we can send you updates.

You will be directed to the online appendices after registering your email.

* Required

Name *

Your answer

Email *

Your answer

SUBMIT

Never submit passwords through Google Forms.

This form was created inside of University of Michigan. Report Abuse - Terms of Service

Google Forms

© 2019 Min L et al. *JAMA Network Open.*

Measurement of Fall Injury with Health Care System Data and Assessment of Inclusiveness and Validity of Measurement Models

Lillian Min, MD MSHS, Mary Tinetti, MD, Kenneth M. Langa, MD, PhD,

Jinkyung Ha, PhD, Neil Alexander, MD, Geoffrey Hoffman, MD

Online Coding Appendices:

eTable 1: E codes indicating fall as cause of injury

eTable 4 and 5: Coding appendices overview and SAS code to analyze Medicare claims for potential injury

eTable 6: SAS code for the fall algorithm 1 (acute care)

eTable 7: SAS code for the fall algorithm 2 (balanced)

eTable 8: SAS code for the fall algorithm 3 (inclusive)

eTable 11: Matching ICD-10 codes for the ICD-9 codes used in the fall algorithms

Citation:

Min L, Tinetti M, Langa KM, Ha J, Alexander N, Hoffman G. Measurement of Fall Injury with Health Care System Data and Assessment of Inclusiveness and Validity of Measurement Models. JAMA Open, 2019

© 2019 Min L et al. *JAMA Network Open.*
